# Dual-targeted engineered mesenchymal stem cell-derived extracellular vesicles delivering Nedd4 attenuate renal fibrosis in diabetic kidney disease

**DOI:** 10.1016/j.mtbio.2026.103097

**Published:** 2026-04-05

**Authors:** Cheng Ji, Bei Li, Jiahui Zhang, Linru Shi, Leiyi Zhang, Hui Shi, Xu Zhang, Wenrong Xu, Lixia Yu, Qifeng Liu, Hui Qian

**Affiliations:** a Department of Nephrology, Affiliated Kunshan Hospital of Jiangsu University, Kunshan, 215300, China; b Jiangsu Key Laboratory of Laboratory Medicine, Department of laboratory Medicine, School of Medicine, Jiangsu University, Zhenjiang, 212013, China

**Keywords:** Diabetic kidney disease fibrosis, Dual-targeted, Engineered extracellular vesicles, Nedd4, Antagonize renal fibrotic niche

## Abstract

Renal interstitial fibrosis is a critical feature of renal injury in diabetic kidney disease (DKD), and the degree of fibrosis progressively worsens as the disease advances. Therefore, understanding the complexity of the renal fibrosis microenvironment is vital for improving anti-fibrotic therapeutic outcomes. In this study, we found that CD68^+^VEGF^+^TGF-β_1_^+^ macrophages promote the overexpression of NUAK family SNF1-like kinase 1 (NUAK1) in tubular epithelial cells, as revealed by single-cell transcriptome sequencing. Mechanistically, increased NUAK1 directly binds to Yes1-associated protein (YAP), disrupts its interaction with large tumor suppressor kinase 1 (LATS1), and promotes YAP nuclear translocation, thereby accelerating the progression of DKD fibrosis. In addition, umbilical cord mesenchymal stem cell-derived extracellular vesicles (MSC-EVs) attenuate renal interstitial fibrosis in DKD by inhibiting the NUAK1/YAP pathway. Analysis of conditional Nedd4 loss-of-function transgenic mice showed that MSC-EVs exert anti-fibrotic effects by delivering the E3 ubiquitin ligase Nedd4, which mediates NUAK1 degradation. Furthermore, we constructed dual-targeted engineered MSC-EVs modified with superparamagnetic nanoparticles and loaded with Nedd4 (SPION-EVs-Nedd4), which improved their targeted anti-fibrotic therapeutic effects. Together, these findings provide a new strategy for the prevention and treatment of DKD interstitial fibrosis, with significant scientific value and clinical translational potential.

## Highlights

• CD68^+^VEGF^+^TGFβ1^+^ macrophages drive NUAK1 upregulation in DKD tubules.
• NUAK1 facilitates DKD fibrosis by disrupts the interaction between YAP and p-LATS1.
• Tubule-specific Nedd4 knockout confirmed MSC-EVs target to counter DKD fibrosis.
• Dual-targeted engineered SPION-EVs-Nedd4 significantly enhances the anti-fibrotic efficacy.

## Graphical abstract

**Figure:**
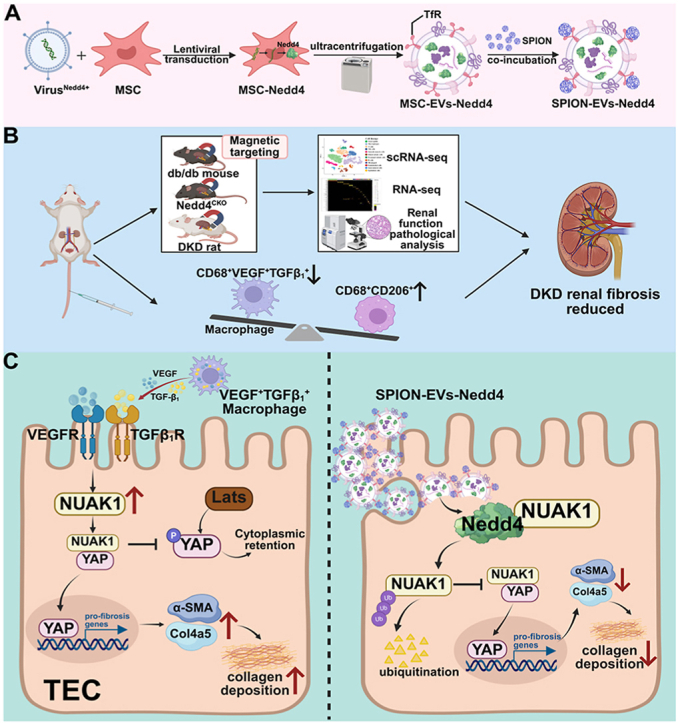

## 1 Introduction

Diabetic kidney disease (DKD) is one of the most common chronic complications of diabetes, and it is primarily characterized by renal interstitial collagen deposition and fibrosis [1,2]. The global prevalence and mortality of DKD are increasing annually, with markedly higher rates in developing countries than in Western developed nations [3,4]. Current DKD treatments focus on pharmacologically controlling blood glucose and improving hemodynamic and metabolic factors [5]. However, there is a lack of safe and effective targeted therapies for inflammation and fibrosis [6]. Thus, developing novel preventive and therapeutic strategies against DKD fibrotic progression is of great significance.

Mesenchymal stem cell-derived extracellular vesicles (MSC-EVs) have emerged as powerful paracrine mediators for tissue repair and regeneration [7,8]. EVs can carry diverse biologically active molecules, including proteins, lipids, and nucleic acids, and transfer them to target cells to regulate their behavior [9,10]. Preclinical studies have demonstrated that MSC-EVs hold promise for mitigating renal injury and fibrosis in various kidney disease models [11]. Our preliminary studies in DKD models showed that MSC-EVs can significantly alleviate renal fibrosis and collagen deposition. Single-cell transcriptomics further revealed their ability to reprogram macrophage-tubular epithelial cell crosstalk in the tubules and downregulate the NUAK family SNF1-like kinase 1 (NUAK1, a serine/threonine kinase involved in fibrotic signaling)/YAP axis. However, the underlying mechanisms of NUAK1 inhibition mediated by MSC-EVs remain unclear, particularly regarding whether it involves direct regulation of NUAK1 protein stability.

Ubiquitination, a pivotal post-translational modification regulated by E3 ubiquitin ligases, plays a critical role in the dynamic stability and function of proteins in cellular homeostasis and disease [12]. Nedd4 (neural precursor cell-expressed developmentally down-regulated protein 4) is a HECT-domain E3 ligase [13]. It can recognize substrates via the WW domain and regulate multiple processes, including inflammation, fibrosis, and cellular stress responses [14,15]. Interestingly, bioinformatics analysis indicates that NUAK1, a key activator of pro-fibrotic YAP signaling, may be a potential substrate of Nedd4, suggesting a ubiquitination-mediated regulatory axis that may be exploited by MSC-EVs [16,17]. However, it remains unclear whether MSC-EVs promote the degradation of NUAK1 in diabetic kidney fibrosis through Nedd4.

EVs possess advantages such as low immunogenicity, excellent biocompatibility, and high drug-loading capacity, making them an emerging drug-delivery vector [18]. Key strategies for engineering MSC-EVs include surface modification and loading therapeutic molecules, which boost their tissue-targeting and delivery efficiency to enhance therapeutic effects [19]. Magnetic targeting therapy, an effective method to enhance targeting specificity, involves modifying biological nanomaterials with magnetic nanoparticles. Under an external magnetic field, these modified materials leverage superparamagnetic properties and magnetic navigation for directed migration to injury sites [20]. Our team previously developed a novel EV-based nanodelivery platform using superparamagnetic nanoparticles [21]. Its success in cancer and kidney therapy offers solid technical and experimental support for engineering MSC-EVs to target DKD fibrosis inhibition [22].

Here, by integrating single-cell transcriptomics, proteomics, and cell-specific Nedd4 knockout models, we reveal the key mechanisms by which MSC-EVs deliver Nedd4 to tubular epithelial cells. These EVs regulate macrophage phenotype transformation and influence the NUAK1/YAP signaling pathway, exerting anti-fibrotic effects and slowing DKD fibrotic progression. We further constructed a dual-targeted delivery system, SPION-EVs-Nedd4. This system combines the innate biological homing capability of MSC-EVs to injured renal tissues with physical magnetic targeting driven by an external magnetic field, thereby enhancing kidney targeting and therapeutic effects. This work not only reveals a new ubiquitination-dependent pathway in DKD fibrosis but also offers a translatable nano-platform for precision anti-fibrotic therapy.

## 2 Results

### 2.1 Single-cell sequencing reveals MSC-EVs induce a macrophages polarization shift in DKD models

Prior to EV isolation, hucMSCs were characterized. Flow cytometry confirmed positive expression of MSC surface markers (CD73, CD105, and CD166) and negative expression of hematopoietic markers (CD11b, CD34, and CD45). Additionally, differentiation assays verified their osteogenic and adipogenic potential (Supplemental Fig. 1). MSC-EVs are nanoscale membrane vesicles that exhibit a typical cup-shaped vesicle structure observed by transmission electron microscopy (TEM) and atomic force microscopy (AFM), with a particle size of approximately 112.9 ± 15.6 nm as detected by nanoparticle tracking analysis (NTA) (Supplemental Fig. 2). In two DKD models, high-fat diet combined with streptozotocin (STZ) and db/db mice, MSC-EVs accumulated in the impaired kidneys, thereby reducing renal injury indicators. The expression levels of α-SMA and Smad2/3 were significantly downregulated, renal interstitial collagen deposition was significantly reduced, delaying the progression of DKD fibrosis (Supplemental Figs. 3 and 4).

**Fig. 1:**
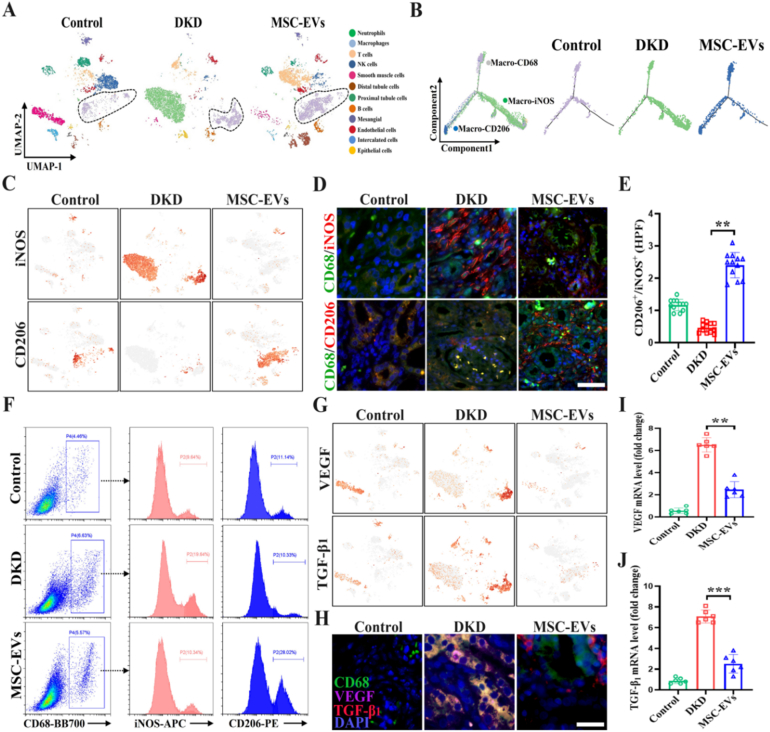
**Macrophage polarization shift in DKD kidneys induced by MSC-EVs.** A. Single-cell RNA-sequencing analysis of kidney cell populations using t-SNE plots; B. Pseudotime analysis of macrophage differentiation trajectories in three groups; C. t-SNE plots of macrophage subtypes; D-E. Immunofluorescence detection and statistical analysis of CD206 and iNOS expression in renal macrophages in three groups (Scale bar: 100 μm); F. Flow cytometry quantification of CD206^+^ and iNOS^+^ macrophage proportions in renal tissues; G. Expression of fibrosis-related genes in macrophages from DKD renal tissue; H. Multicolor immunofluorescence detection of CD68^+^VEGF^+^TGF-β_1_^+^ expression in macrophages (Scale bar: 100 μm); I-J. qRT-PCR detection of VEGF^+^TGF-β_1_^+^ mRNA changes in renal tissue (n = 6). All values are presented as the mean ± SD. ∗∗*P* < 0.01, ∗∗∗*P* < 0.001.

To investigate the renal repair effects of MSC-EVs, we performed single-cell RNA sequencing on renal tissues from the Control, DKD, and DKD + MSC-EVs groups; the overall experimental design and analytical workflow are summarized in Supplemental Fig. 5. Based on cell cluster marker genes, kidney tissues were classified into 12 populations: neutrophils, macrophages, T cells, NK cells, smooth muscle cells, distal tubule cells, proximal tubule cells, B cells, mesangial cells, interstitial cells, endothelial cells, and epithelial cells **(**Supplemental Figs. 6–8**)**. Single-cell sequencing revealed significant macrophage polarization and clustering in the DKD group, while the MSC-EVs group's clustering pattern resembled the control group (Fig. 1A). Pseudotime analysis and t-SNE plots of community gene expression demonstrated that renal macrophages in the DKD group exhibited increased iNOS^+^ populations, whereas CD206^+^ populations dominated in the MSC-EVs group (Fig. 1B and C). Immunofluorescence and flow cytometry confirmed elevated CD68^+^iNOS^+^ macrophages in DKD versus predominant CD68^+^CD206^+^ macrophages in the MSC-EV group. The CD206^+^/iNOS^+^ ratio was significantly higher after MSC-EVs treatment, confirming macrophage polarization shift (Fig. 1D–F). Further analysis revealed significant overexpression of fibrotic genes (VEGF, TGF-β_1_) in DKD macrophages, which were downregulated following MSC-EVs intervention (Fig. 1G). Multicolor immunofluorescence and qRT-PCR showed CD68^+^VEGF^+^TGF-β_1_^+^ macrophage recruitment in DKD kidneys (Supplemental Fig. 9), with significant reduction post MSC-EVs intervention (Fig. 1H and J). These findings suggested MSC-EVs modulate renal fibrosis through induce macrophage polarization.

### 2.2 NUAK1 disrupts the interaction between YAP and p-LATS1 kinases to promote DKD renal fibrosis

**Fig. 2:**
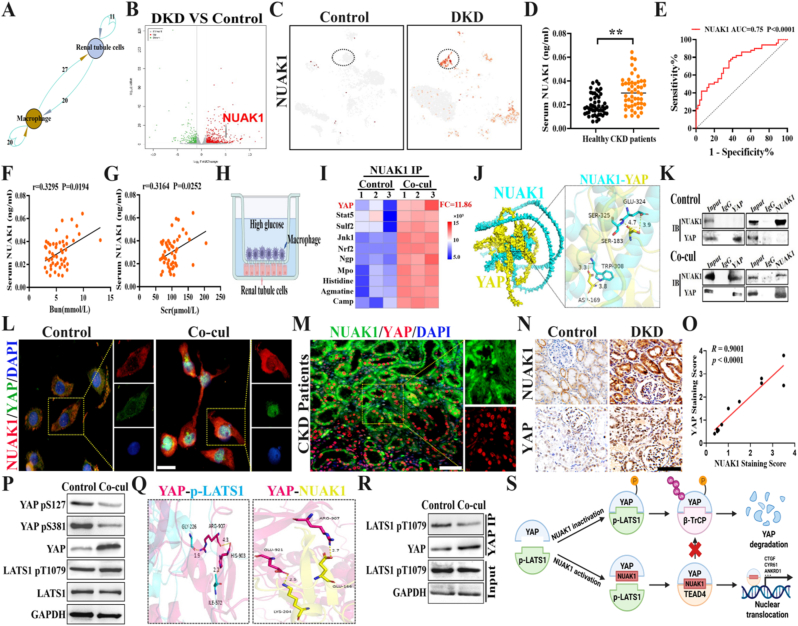
**NUAK1 disrupts the interaction between YAP and p-LATS1 kinases to promote renal fibrosis.** A. Number of signaling pathways in the interaction between macrophages and renal tubular epithelial cells; B. Top gene expression profile in renal tissues of the DKD and control groups; C. t-SNE plot of NUAK1 expression in renal tubular epithelial cells; D. Relative serum NUAK1 levels in patients with DKD and healthy controls (n = 50); E. Receiver operating characteristic curve of the performance of serum NUAK1 levels in diagnosing DKD among 50 patients. The AUROC is shown; F-G. Correlation of serum NUAK1 levels with blood urea nitrogen (BUN) and serum creatinine (Scr) levels; H. Schematic diagram of co-culture model between macrophages and NRK-52E cells; I. NRK-52E cells were co-cultured with macrophages for 48 h, and cell lysates were immunoprecipitated using an anti-NUAK1 antibody. Metabolites in the immunocomplexes were extracted. The quantitative abundance of metabolites was measured using a trace-level metabolite detection method based on liquid chromatography-mass spectrometry (LC-MS) (n = 3); J. Docking model of NUAK1 and YAP. The full-length NUAK1 structure was predicted using AlphaFold. Surface presentation of the NUAK1 (cyan) with YAP (yellow) bound to its central cavity; K. Co-immunoprecipitation to detect the interaction between NUAK1 and YAP; L. Immunofluorescence staining detects the expression of NUAK1/YAP in NRK-52E cells stimulated by macrophage co-culture (Scale bar: 100 μm); M. Immunofluorescence staining detects the co-localization of NUAK1/YAP in renal tissue of chronic kidney disease patients (Scale bar: 100 μm); N. Representative IHC staining images of NUAK1 and YAP in the control group and the DKD group; O. Correlation analysis of IHC staining scores between NUAK1 and YAP; P. NRK-52E cells were co-cultured with macrophages for 48 h, and cell lysates were immunoblotted using the indicated antibodies; Q. Docking models of YAP-p-LATS1 and YAP-NUAK1; R. NRK-52E were co-culture with macrophage for 48 h, and cell lysates were immunoprecipitated using anti-YAP antibody, followed by blotting with the indicated antibodies; S. A diagram summarizing the proposed model in which NUAK activation disrupts the YAP and p-LATS1 interaction, increasing YAP stability and nuclear expression, thereby promoting renal fibrosis. All values are presented as the mean ± SD. ∗∗*P* < 0.01. (For interpretation of the references to color in this figure legend, the reader is referred to the Web version of this article.)

Previous single-cell sequencing results demonstrated that VEGF^+^TGF-β_1_^+^ macrophage activation in DKD. Further analysis of the signaling pathways mediating the interaction between macrophages and renal tubular epithelial cells within DKD using cell interaction revealed the existence of 47 pairs of pathways (Fig. 2A). To identify specific signaling pathways mediating pro-fibrotic effects, we performed transcriptomic sequencing of renal tissues. The results revealed 498 upregulated and 221 downregulated genes in DKD proximal tubular epithelial cells versus normal controls. GEO analysis of the GSE200322, 262687,190205 dataset revealed a 3.24-fold increase in the mRNA level of NUAK1 in the renal tubules of DKD patients (Supplemental Fig. 10). In the DKD model, VEGF^+^TGF-β_1_^+^ macrophages were markedly enriched, secreting fibrogenic factors that induced NUAK1 overexpression in tubular epithelial cells (Fig. 2B and Supplemental Fig. 11). t-SNE plots of community gene expression confirmed NUAK1 high-expression in DKD tubular cells (Fig. 2C).

To further validate the clinical correlation between NUAK1 and DKD progression, we compared serum NUAK1 levels between 50 patients with DKD and 50 healthy individuals. The results revealed that the NUAK1 level in the serum of DKD patients was significantly higher than that of healthy controls (Fig. 2D). The AUROC analysis demonstrated that serum NUAK1 had a diagnostic efficiency of 0.75 for DKD (Fig. 2E). Furthermore, serum NUAK1 showed moderate correlations with blood urea nitrogen (BUN) (r = 0.3295, p = 0.0194) and serum creatinine (Scr) and (r = 0.3164, p = 0.0252) (Fig. 2F and G).

To reveal the internal interaction mechanism in DKD, we co-cultured NRK-52E with macrophages in a high-glucose environment for 48 h (Fig. 2H). Thus, we identified small-molecule metabolites that directly bind to NUAK1 using the trace-level metabolite quantitation. Under high-glucose macrophage co-culture conditions, NRK-52E cells exhibited enhanced NUAK1–YAP interaction, as demonstrated by co-immunoprecipitation analysis. In parallel, trace-level metabolite quantitation identified marked alterations in NUAK1-associated small-molecule metabolites (Fig. 2I and Supplemental Fig. 12). To explore the specific mechanism of protein-protein interaction (PPIs) between NUAK1 and YAP, molecular docking analysis further showed that the indole amino group of TRP-308 in NUAK1 forms a 3.8 Å-interaction force with ASP-169 in YAP, and forms a 3.3 Å-hydrogen bond with the carboxyl group of its side chain. The main chain carbonyl oxygen of SER-183 in YAP forms a 4.7 Å-ion bond with the main chain primary amino group of SER-325 in NUAK1, and forms a 3.9 Å-hydrogen bond with the side chain carboxyl group of GLU-324 in NUAK1 (Fig. 2J). Co-immunoprecipitation results showed that YAP directly interacted with NUAK1 (Fig. 2K). The immunofluorescence results showed significantly increased expression of cytoplasmic NUAK1 (red) and nuclear YAP (green) in renal tubular cells after macrophage stimulation (Fig. 2L).

To verify clinical relevance, we analyzed renal biopsy specimens from CKD patients at the affiliated hospital. Immunofluorescence staining results confirming the activation of the NUAK1/YAP pathway is involved in the progression of DKD patients (Fig. 2M). Immunohistochemical staining observed that the expression levels of NUAK1 and YAP were significantly higher in DKD tissues than in control tissues, and YAP was predominantly localized in the nuclei of DKD patients tissues (Fig. 2N). The scatter plot revealed a strong positive correlation between NUAK1 and YAP staining scores (R = 0.9901, p < 0.0001), indicating that both are up-regulated and closely linked in DKD (Fig. 2O).

YAP is an important downstream effector molecule of the Hippo pathway and is phosphorylated by the MST1/2 and large tumor suppressor kinases 1 and 2 (LATS1/2) cascades(*24, 25*). Next, we aimed to answer how NUAK1 activation impacts the interaction of YAP and p-LATS1. Small-molecule metabolites can directly bind to target proteins and act as functional ligands to regulate protein-protein interactions and influence protein activity(*26*). Therefore, we evaluated the effect of NUAK1 activation on the Hippo pathway. First, NUAK1 activation inhibited YAP phosphorylation and increased YAP protein abundance, whereas the protein abundance and phosphorylation of crucial kinase LATS1 was barely affected (Fig. 2P and Supplemental Fig. 13). Interestingly, we observed that ARG-907, one of the predominant amino acids of the YAP protein interacting with NUAK1, occupies the partial binding sites of YAP and p-LATS1, suggesting that NUAK1 may competitively bind to YAP alongside p-LATS1 (Fig. 2Q). This led us to find that NUAK1 may participate in the regulation of the interaction between YAP and p-LATS kinases by binding to YAP. Importantly, we observed that NUAK1 activation markedly inhibited the interaction between YAP and phosphorylated LATS1 (p-LATS1) (Fig. 2R and Supplemental Fig. 14). These results suggested that NUAK1 activation inhibits YAP protein phosphorylation mainly by impacting the interaction of YAP and p-LATS1, rather than by affecting the phosphorylation of the crucial kinase of the Hippo pathway (Fig. 2S).

### 2.3 MSC-EVs exhibit anti-fibrotic effects by inhibiting the NUAK1/YAP pathway

**Fig. 3:**
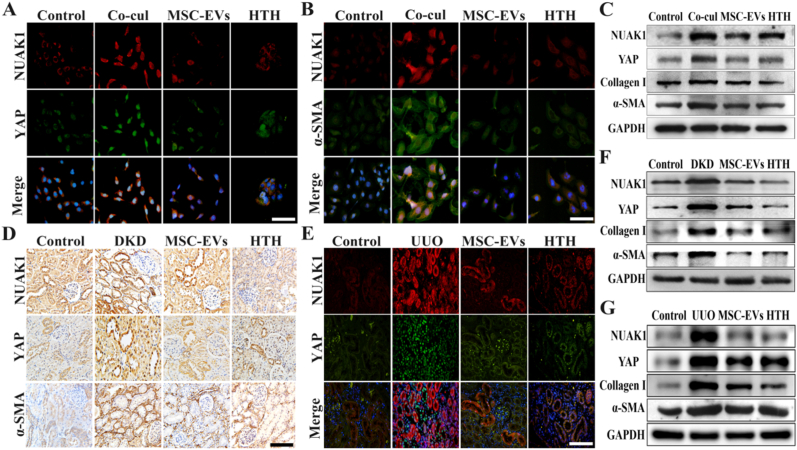
**MSC-EVs exhibit anti-renal fibrosis effects by inhibiting the NUAK1/YAP pathway.** A. Representative immunofluorescence staining images of NUAK1 and YAP in NRK-52E after co-culture with macrophages (Scale bar: 100 μm); B. Representative immunofluorescence staining images of NUAK1 and α-SMA in NRK-52E after co-culture with macrophage (Scale bar: 100 μm); C. Western blot to detect the expression of fibrosis-related proteins in vitro; D. Immunohistochemistry staining to detect protein expression after MSC-EVs intervention in the DKD model (Scale bar: 100 μm); E. Immunofluorescence staining detects NUAK1/YAP expression in the UUO model after intervention with MSC-EVs and HTH (Scale bar: 100 μm); F-G. Western blot to detect the expression of fibrosis-related proteins in DKD model and UUO model.

Previous studies have confirmed the crucial role of NUAK1/YAP pathway activation in the progression of renal fibrosis in DKD. The community gene t-SNE map of single-cell sequencing revealed that NUAK1 in renal tubules was significantly downregulated in the MSC-EVs group (Supplemental Fig. 15). Immunofluorescence analysis revealed downregulated NUAK1 expression, nuclear-to-cytoplasmic translocation of YAP, and reduced α-SMA levels in HTH (HTH-01-015, a selective NUAK1 inhibitor)-treated NRK-52E cells. MSC-EVs recapitulated these effects (Supplemental Fig. 16), suggesting that NUAK1 inhibition underlies the therapeutic mechanism of action (Fig. 3A and B). These observations were confirmed by Western blot analysis (Fig. 3C and Supplemental Fig. 17). In vivo, we established renal fibrosis models DKD and unilateral ureteral obstruction (UUO). Immunohistochemistry and tissue protein analysis demonstrated that tubular NUAK1 overexpression promoted YAP nuclear translocation and subsequent α-SMA expression in DKD model (Fig. 3D). MSC-EVs treatment significantly reduced both NUAK1 and YAP levels (Fig. 3E–G, Supplemental Figs. 18 and 19). These findings demonstrate that MSC-EVs attenuate renal fibrosis through NUAK1/YAP pathway.

### 2.4 MSC-EVs deliver E3 ubiquitin ligase Nedd4 to impact NUAK1 protein stability

**Fig. 4:**
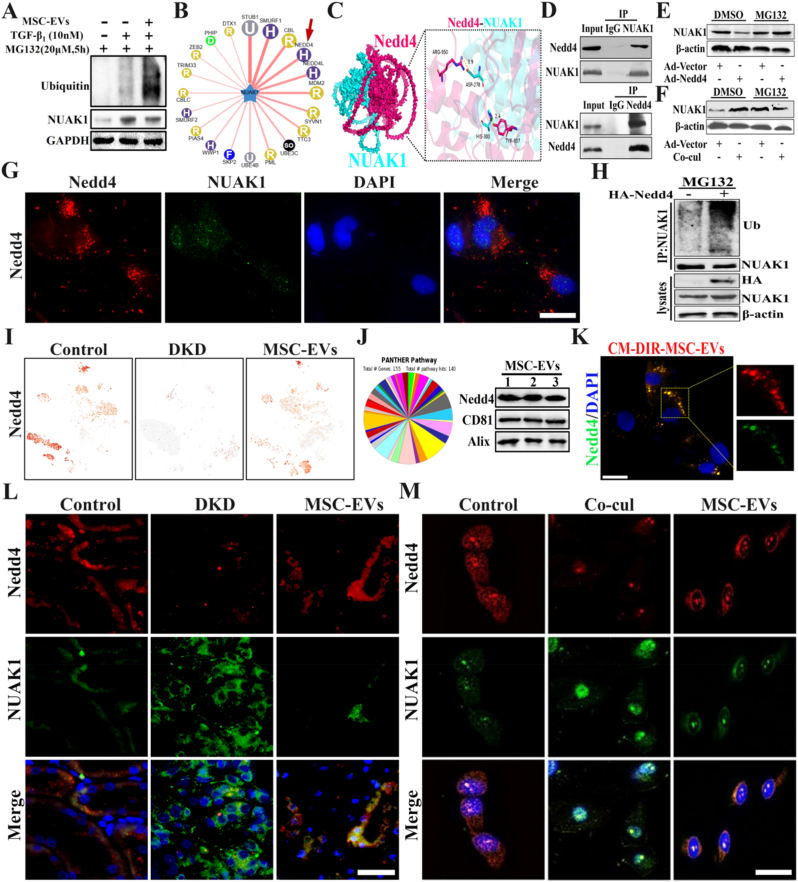
**MSC-EVs deliver NEDD4 to mediate ubiquitylation and degradation of NUAK1 via direct binding.** A. Co-immunoprecipitation to detect ubiquitin bound to NUAK1 after MSC-EVs intervention with MG132 treatment; B. The E3 ubiquitin ligases acting on NUAK1 were predicted with the online database UbiBrowser, an integrated bioinformatics platform. The capital letters in dots indicate the initial letters of E3 ubiquitin ligases-domains: F refers to F-box domain, R refers to RING domain, H refers to HECT domain, U refers to UBOX domain. The predicted interactions are arranged in descending order clockwise based on the confidence score; C. Left panel: magnified view of NUAK1-Nedd4 binding. Right panel: The pyrrolidine ring of NUAK1 (HIS-303) forms a hydrogen bond with the hydroxyl group of the side chain of Nedd4 (TYR-937),both have a cyclic rigid planar structure and there is a π-π stack. The secondary amino group of the Nedd4 side chain (ARG-950) forms a hydrogen bond with the carboxyl group of NUAK1 (ASP-278); D. Parental NRK-52E cell lysates were co-immunoprecipitated with anti-NUAK1, anti-Nedd4 antibodies, and Nedd4 binding to NUAK1 was examined with WB analysis; E. Representative western blots of indicated proteins in NRK-52E transfected with Ad-Vector or Ad-Nedd4 (left) after DMSO or MG132 treatment; F. Representative western blots of indicated proteins in NRK-52E transfected with Ad-Vector or Co-culture with macrophages for 24 h (right), with DMSO or MG132 treatment; G. Immunofluorescence staining showed the colocalization of Nedd4 (red) and NUAK1 (green) within cells; H. Representative Western blots of indicated proteins after IP with anti-HA affinity gel and Western blots of indicated proteins in the cell lysates of 293T cells transfected with the indicated plasmids, with MG132 treatment; I. Single-cell sequencing analysis of Nedd4 expression distribution in three groups; J. Proteomics and Western blot detection of Nedd4 expression in MSC-EVs; K. Immunofluorescence staining detects Nedd4 expression in NRK-52E cells after uptake of MSC-EVs (Scale bar: 20 μm); L. Immunofluorescence staining detects Nedd4/NUAK1 expression (Scale bar: 100 μm); M. Immunofluorescence staining detects Nedd4 and NUAK1 expression after MSC-EVs intervention (Scale bar: 50 μm). (For interpretation of the references to color in this figure legend, the reader is referred to the Web version of this article.)

Previous studies confirmed MSC-EVs significantly inhibit NUAK1 expression. Treatment of renal tubular epithelial cells with the proteasome inhibitor MG132 revealed increased ubiquitin-bound NUAK1 following MSC-EVs intervention, suggesting protein stability regulation mediates this effect (Fig. 4A). Analysis using the UbiBrowser database showed that NUAK1 is highly connected with the E3 ubiquitin ligase Nedd4 (Fig. 4B). Further molecular docking simulations were conducted to investigate the protein-protein interaction binding sites between NUAK1 and Nedd4 (Fig. 4C and Supplemental Fig. 20). Co-immunoprecipitation assays confirmed that NUAK1 could interact with Nedd4 (Fig. 4D). Western blotting confirmed that Nedd4 overexpression downregulated NUAK1. In MG132-treated cells, NUAK1 expression rebounded significantly. This indicates MG132 blocked the protein degradation pathway, stabilizing NUAK1 which would otherwise be degraded by Nedd4 (Fig. 4E, Supplemental Fig. 21). In DMSO treated NRK-52E cells, macrophage co-culture promoted increased NUAK1 expression. In MG132-treated cells, this regulatory mechanism was disrupted, reducing NUAK1 expression (Fig. 4F, Supplemental Fig. 22). Immunofluorescence staining visualized the intracellular colocalization of Nedd4 and NUAK1 (Fig. 4G). Co-immunoprecipitation assays showed that with MG132 present, NUAK1 ubiquitination rose but its protein level fell following HA-Nedd4 treatment. This confirms that the E3 ubiquitin ligase Nedd4 mediates NUAK1 ubiquitination and degradation (Fig. 4H). To further assess whether Nedd4 promotes NUAK1 ubiquitination in an E3 ligase activity-dependent manner, we performed a cell-based ubiquitination assay using wild-type and catalytically inactive Nedd4 constructs. Cells were co-transfected with Flag-NUAK1, His-Ub, and either empty vector, HA-Nedd4-WT, or HA-Nedd4 mutant, followed by MG132 treatment before harvest. After immunoprecipitation of Flag-NUAK1, immunoblotting with anti-His revealed that wild-type Nedd4 increased NUAK1 ubiquitination, whereas the catalytically inactive mutant showed a much weaker effect **(**Supplemental Fig. 23**).** These data support a role for Nedd4 in promoting NUAK1 ubiquitination in an E3 ligase activity-dependent manner.

Single-cell RNA sequencing demonstrated significant alterations in ubiquitin ligase pathways, with Nedd4 downregulation in DKD tubular cells that MSC-EVs restored (Fig. 4I). LC-MS/MS and Western blot confirmed Nedd4 presence in MSC-EVs (Fig. 4J). CM-DiR (1,1′-dioctadecyl-3,3,3′,3′-tetramethylindotricarbocyanine iodide) -labeled MSC-EVs incubated with NRK-52E cells for 24 h showed intracellular Nedd4 elevation co-localizing with MSC-EVs (Fig. 4K). Immunofluorescence revealed suppressed tubular Nedd4 in DKD kidneys, correlating with NUAK1 up-regulation. MSC-EVs restored Nedd4 while inhibiting NUAK1, a finding replicated in vitro (Fig. 4L and M). Together, these data suggest that MSC-EVs delivered Nedd4 to promote the degradation of NUAK1.

### 2.5 The anti-fibrotic effect of MSC-EVs was weakened in Nedd4 knockout mice

**Fig. 5:**
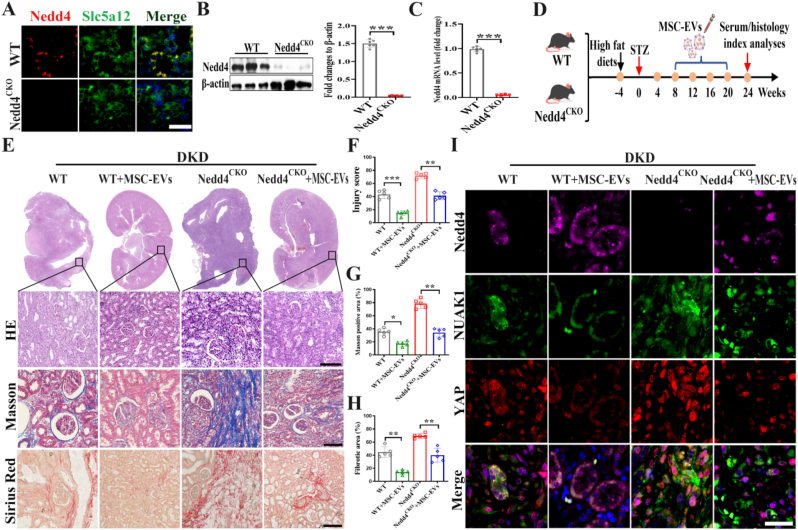
**Knockout of the Nedd4 gene in renal tubular epithelial cells attenuates the anti-fibrotic efficacy of MSC-EVs.** A. Immunofluorescence staining detects the expression of Nedd4 in renal tissue (Scale bar: 100 μm); B. Western blot to detect and statistically analyze the expression of Nedd4 in renal tissue; C. qRT-PCR to detect the mRNA expression of Nedd4 in renal tissue; D. Schematic diagram of MSC-EVs intervention in Nedd4^CKO^ mice; E. Histopathological analysis of renal tissue using H&E, Sirius red and Masson's trichrome staining (Scale bar: 100 μm); F-H. Statistical analysis of renal injury scores, Masson's positive ratios, and fibrosis scores (n = 6); I. Immunofluorescence staining detects the expression of Nedd4/NUAK1/YAP in renal tissue (Scale bar: 100 μm). All values are presented as the mean ± SD. ∗*P* < 0.05, ∗∗*P* < 0.01, ∗∗∗*P* < 0.001. (For interpretation of the references to color in this figure legend, the reader is referred to the Web version of this article.)

To determine whether MSC-EVs exert anti-fibrotic effects via Nedd4 delivery, we generated renal tubular epithelial cell-specific Nedd4 knockout mice using the Cre-loxP system. Immunofluorescence, western blot and qRT-PCR confirmed successful Nedd4 knockout in mouse model (Fig. 5A–C, Supplemental Fig. 24). The control group and Nedd4 knockout mice were induced DKD model by intraperitoneal injection of Streptozotocin (STZ), and intervened by intravenous administration of MSC-EVs. (Fig. 5D). HE, Masson's trichrome and Sirius red staining revealed more severe tubular injury and fibrosis in Nedd4 knockout versus wild-type mice. While MSC-EVs alleviated injury and fibrosis in wild-type mice, their therapeutic efficacy was attenuated in knockout mice (Fig. 5E). Injury scores and fibrosis-positive area quantification validated these findings (Fig. 5F–H). Immunofluorescence demonstrated that Nedd4 knockout promoted NUAK1-mediated YAP nuclear translocation, whereas MSC-EVs upregulated tubular Nedd4 expression and suppressed the NUAK1/YAP pathway (Fig. 5I). These results confirm that Nedd4 is the key target protein of MSC-EVs in inhibiting renal fibrosis in DKD.

### 2.6 Biological characterization of dual-targeted engineered SPION-EVs-Nedd4

**Fig. 6:**
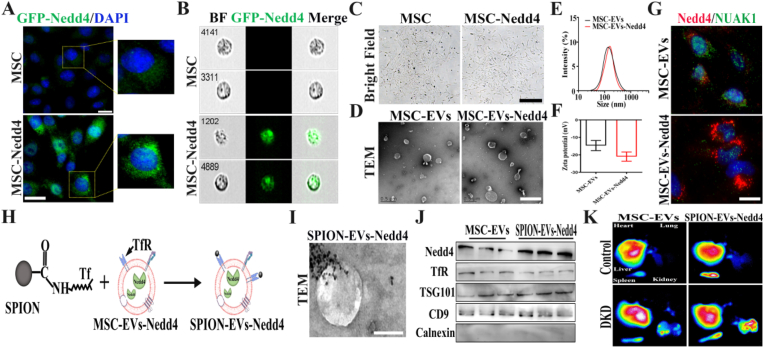
**Preparation and in vivo tracking of dual-targeted engineered SPION-EVs-Nedd4.** A. Immunofluorescence staining for Nedd4 expression in MSCs after transfection (Scale bar: 20 μm); B. Imaging flow cytometry analysis for Nedd4 expression; C. Morphology of MSCs after transfection (Scale bar: 200 μm); D. Transmission electron microscopy images of MSC-EVs and MSC-EVs-Nedd4; E-F. Nanoparticle tracking analysis for size and zeta potential of MSC-EVs-Nedd4; G. Immunofluorescence staining for Nedd4/NUAK1 expression in NRK-52E cells treated with MSC-EVs-Nedd4 (Scale bar: 20 μm); H. Schematic diagram for the preparation of engineered SPION-EVs-Nedd4; I. TEM observation of SPION-EVs-Nedd4 morphology (Scale bar: 100 nm); J. Western blot analysis for surface marker proteins of SPION-EVs-Nedd4; K. In vivo imaging of organ distribution of SPION-EVs-Nedd4 using a small animal in vivo imaging system. Quantitative radiant efficiency of major organs is shown in Supplemental Fig. 28.

Given Nedd4's pivotal role in NUAK1 regulation and renal fibrosis, we constructed a GFP-labeled lentiviral vector carrying the Nedd4 gene to transfect MSCs, aiming to enhance MSC-EVs anti-fibrotic efficacy. Immunofluorescence and imaging flow cytometry confirmed unaffected MSC viability with significant cytoplasmic Nedd4 overexpression (Fig. 6A–C). Ultracentrifugation-isolated EVs exhibited characteristic cup-shaped morphology under TEM, with nanoparticle tracking analysis showing slightly increased size and zeta potential in MSC-EVs-Nedd4 versus MSC-EVs (Fig. 6D–F). Co-culture with renal tubular epithelial cells demonstrated elevated Nedd4 expression and significantly reduced NUAK1 in MSC-EVs-Nedd4-treated cells compared to MSC-EVs controls, confirming enhanced regulatory capacity through Nedd4 overexpression (Fig. 6G, Supplemental Fig. 25).

To enhance renal targeting and anti-fibrotic efficacy of MSC-EVs, dual-targeted engineered SPION-EVs-Nedd4 by conjugating transferrin (Tf)-modified superparamagnetic iron oxide nanoparticles to Tf receptors on MSC-EVs-Nedd4 (Fig. 6H, Supplemental Fig. 26). Transmission electron microscopy showed the morphology of SPION-EVs-Nedd4 (Fig. 6I). Combined with the observed changes in particle size/zeta potential, Western blot characterization of EV markers and TfR, and the enhanced magnetic targeting in vivo, these results collectively support the successful construction of SPION-EVs-Nedd4 (Fig. 6J, Supplemental Fig. 27). For renal targeting evaluation, DIR-labeled SPION-EVs-Nedd4 were intravenously administered to DKD rats. For renal targeting evaluation, CM-DiR-labeled SPION-EVs-Nedd4 were intravenously administered to DKD rats, and major organs were collected at 24 h for ex vivo fluorescence imaging and biodistribution quantification. Small-animal imaging demonstrated significantly enhanced renal signal intensity under external magnetic field guidance, and quantitative analysis of radiant efficiency further confirmed preferential kidney accumulation relative to other major organs (Fig. 6K and Supplemental Fig. 28). Collectively, these findings confirm the successful construction of the dual-targeted engineered SPION-EVs-Nedd4 with SPION modification over-expression Nedd4, laying the foundation for the next step of anti-fibrotic efficacy evaluation.

### 2.7 Dual-targeted engineered SPION-EVs-Nedd4 significantly enhance the anti-fibrotic effect

**Fig. 7:**
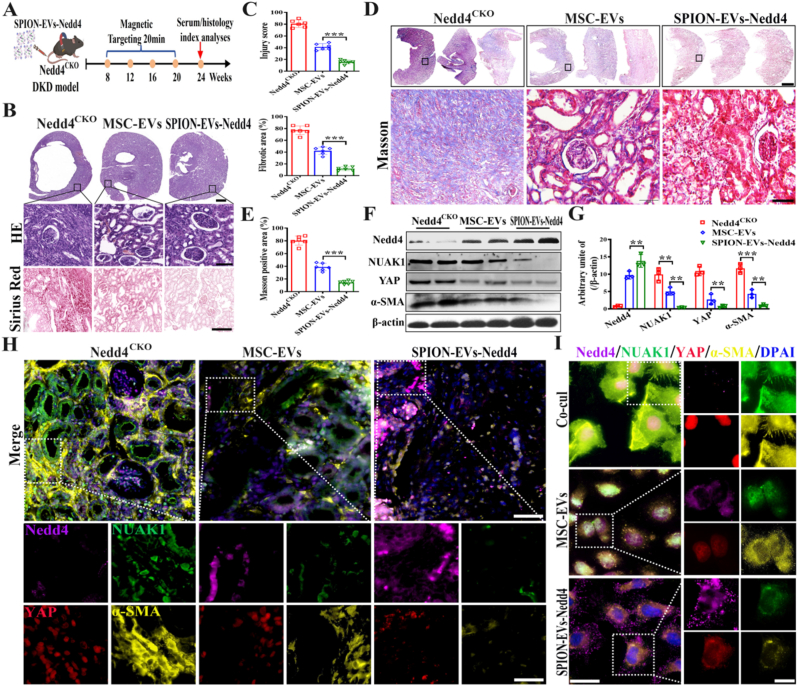
**Dual-targeted engineered SPION-EVs-Nedd4 significantly enhances the anti-fibrotic effects in the kidney.** A. Schematic diagram of SPION-EVs-Nedd4 intervention in Nedd4 gene knockout mice; B. Histopathological HE staining and Sirius red staining of renal tissue (Scale bar: 100 μm); C. Statistical analysis of renal injury scores and fibrosis scores in six groups (n = 6); D. Masson's trichrome staining of renal tissue (Scale bar: 100 μm); E. Statistical analysis of Masson's positive ratios in six groups (n = 6); F. Western blot to detect the expression of related proteins; G. Statistically analyze the expression in renal tissues (n = 3); H. Immunofluorescence staining detects the expression of Nedd4/NUAK1/YAP/α-SMA in renal tissue (Scale bar: 100 μm); I. Immunofluorescence staining detects Nedd4/NUAK1/YAP/α-SMA expression after SPION-EVs-Nedd4 intervention (Scale bar: 50 μm). All values are presented as the mean ± SD. ∗∗*P* < 0.01, ∗∗∗*P* < 0.001. (For interpretation of the references to color in this figure legend, the reader is referred to the Web version of this article.)

To evaluate the efficacy of engineered SPION-EVs-Nedd4 in DKD, we administered to DKD mouse model with tubular epithelial cell-specific Nedd4 gene knockout and applied an external magnetic field to assess its anti-fibrotic effects (Fig. 7A). Histopathological examination via HE and Sirius Red staining showed that, compared to the MSC-EVs group, SPION-EVs-Nedd4 treatment more effectively improved kidney structure in Nedd4-knockout mice and reduced tubular vacuolar degeneration and collagen deposition (Fig. 7B and C). Masson's trichrome staining further confirmed the anti-fibrotic effect of SPION-EVs-Nedd4. The treatment group showed markedly reduced collagen deposition, and quantitative analysis of Masson's trichrome staining positivity supported this finding (Fig. 7D and E). Mechanistically, Western blot of kidney tissue showed that SPION-EVs-Nedd4 restored Nedd4 expression, downregulated NUAK1/YAP, and reduced α-SMA expression (Fig. 7F and G, Supplemental Fig. 29). Renal immunofluorescence further confirmed that SPION-EVs-Nedd4 delivered Nedd4 to tubular epithelial cells, induced degradation of NUAK1, blocks the nuclear entry of YAP, decreased α-SMA, and achieved an enhanced anti-fibrotic cascade effect (Fig. 7H). In vitro model where macrophages and NRK-52E cells were co-cultured under high-glucose stimulation and treated with SPION-EVs-Nedd4, the results were consistent with in-vivo findings, showing a significant reversal of fibrotic changes in tubular epithelial cells (Fig. 7I). To verify the stability and safety of SPION-EVs-Nedd4, the changes in liver, kidney and heart function indicators were detected by a biochemical analyzer. Compared with the MSC-EVs group, the related indicators of SPION-EVs-Nedd4 were down-regulated (Supplemental Fig. 30). In vivo, HE staining of pathological tissues revealed that SPION-EVs-Nedd4 had no effect on the structure of heart, liver, spleen and lung tissues and did not change the tissue structure, indicating the safety of SPION-EVs-Nedd4 in vivo. (Supplemental Fig. 31). In addition, long-term biosafety was further evaluated by monitoring body weight and renal function during treatment. No significant differences in body weight, serum creatinine, or BUN were observed after administration of engineered EVs, indicating that the treatment was well tolerated and did not cause overt systemic or renal toxicity **(**Supplemental Fig. 32**).** In summary, these experimental results validate that the modified dual-targeted engineered SPION-EVs-Nedd4, under the enhancement of an external magnetic field, increases the expression of Nedd4 in the kidneys, maximizing the ubiquitination and degradation of NUAK1, achieving a dual-target synergistic effect and further amplifying the anti-fibrotic effect.

**Fig. 8:**
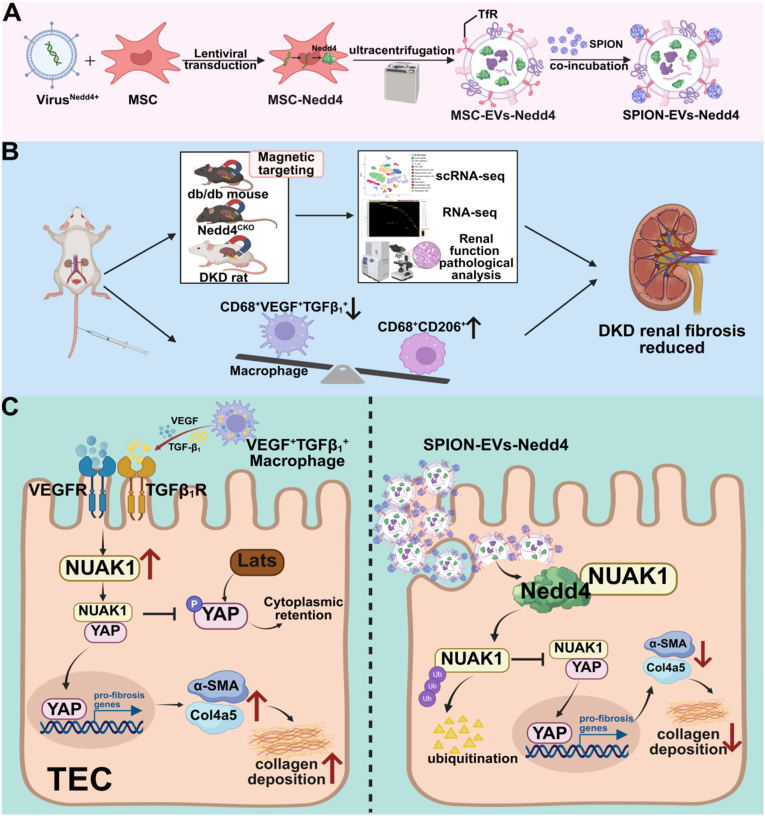
**Dual-targeted engineered MSC extracellular vesicles targeted delivery of Nedd4 to antagonize DKD fibrosis.** A. Construction of dual-targeted engineered SPION-EVs-Nedd4 modified with superparamagnetic nanoparticles loaded with Nedd4; B. The anti-fibrotic effect of dual-targeted engineered nanovesicles was verified by DKD rat models, db/db mouse models and Nedd4 knockdown mice; C. VEGF^+^TGF-β1^+^ macrophages promote the high expression of NUAK1, and NUAK1 disrupts the interaction between YAP and p-LATS1 to promote DKD renal fibrosis, dual-targeted engineered SPION-EVs-Nedd4 facilitate Nedd4 enrichment and delivery, thereby promoting NUAK1 degradation and augmenting anti-fibrotic effects.

In summary, our study demonstrates that CD68^+^VEGF^+^TGF-β_1_^+^ macrophages promote the high expression of NUAK1 in DKD tubular epithelial cells, directly binding to YAP, disrupting its interaction with LATS1, promoting nuclear translocation of YAP, and accelerating the progression of DKD fibrosis. In contrast, hucMSC-EVs regulate macrophage-tubular epithelial cell crosstalk, delivered the E3 ubiquitin ligase Nedd4 and mediated NUAK1 degradation, and ameliorates DKD fibrosis via inhibition of the NUAK1/YAP signaling pathway. In addition, dual-targeted engineered SPION-EV-Nedd4 was constructed by modifying superparamagnetic iron oxide nanoparticles to achieve targeted enrichment and delivery Nedd4 to the kidney tubule cells, significantly improve the anti-fibrotic effect and provide a new therapeutic strategy for DKD (Fig. 8).

## 3 Discussion

This study investigates the role and mechanisms of MSC-EVs in DKD interstitial fibrosis, yielding significant findings. We provide mechanistic insights into the process by which MSC-EVs reprogram macrophage-tubular epithelial cell interactions. Furthermore, we demonstrate that Nedd4-mediated protein homeostasis is a key control point in renal fibrosis. MSC-EVs deliver the E3 ubiquitin ligase Nedd4 to tubular cells, regulating macrophage-tubular epithelial cell crosstalk. They promote NUAK1 ubiquitination and degradation, blocking the NUAK1/YAP pathway and reducing DKD interstitial fibrosis. Moreover, we developed dual-targeted engineered SPION-EVs-Nedd4 with superparamagnetic nanoparticles for better kidney targeting and therapeutic effects, offering new treatment targets and strategies for DKD.

As a cell-free therapeutic strategy, MSC-EVs avoid the immune rejection and tumorigenic risks associated with stem cell transplantation [23]. In recent years, MSC-EVs have drawn widespread attention for their therapeutic effects in acute and chronic kidney injury, and hold great promise for future clinical applications [24]. Here, we first confirmed by various experimental methods that MSC-EVs can effectively reduce the degree of interstitial fibrosis in DKD models. In animal models, MSC-EVs treatment significantly reduced serum creatinine and urea nitrogen levels, improved the pathological structure of kidney tissues, and decreased tubular vacuolar degeneration and interstitial collagen deposition. These results indicate that MSC-EVs possess substantial renal-repair capacity and offer new experimental evidence for DKD treatment.

Single-cell sequencing combined with spatial multi-omics was used to study the distinct phenotypes of glomerular cells, immune cells, tubular epithelial cells, and fibrotic cells in the renal microenvironment of patients with DKD, providing a more reliable basis for prognostic diagnosis [25]. To reveal immune microenvironment changes during MSC-EVs intervention, single-cell transcriptomic sequencing with pseudotemporal trajectory analysis was applied. Our findings demonstrate that MSC-EVs induce renal macrophage polarization, shifting macrophages from the pro-inflammatory M1 (iNOS^+^) to the anti-inflammatory M2 (CD206^+^) type. This macrophage phenotype switching helps reduce inflammation, suppress fibrotic gene expression and slow interstitial fibrosis progression [26]. Although a direct macrophage-intrinsic effect of EV-delivered Nedd4 cannot be completely excluded, our current data more strongly support an indirect mechanism whereby Nedd4 acts primarily in tubular epithelial cells to alleviate injury and fibrotic stress, thereby remodeling the renal microenvironment and favoring reparative macrophage polarization.

Consistent with this interpretation, transcriptomic sequencing analysis of gene expression profiles showed changes in the expression of NUAK1 in tubular epithelial cells between the DKD group and the MSC-EVs group. Studies have shown that NUAK1, a protein kinase regulating cell metabolism, migration, and cell cycle progression, is expressed in mouse models and human tissues via TGF-β1 induction. NUAK1 activates the YAP and Smad pathways, forming a positive feedback loop that promotes fibrosis [17]. Cellphone DB analysis revealed multiple interaction pathways between macrophages and tubular epithelial cells. In DKD, activated VEGF^+^TGF-β_1_^+^ macrophages induce NUAK1 over-expression in tubular epithelial cells, activating the YAP pathway and enhancing the expression of fibrosis-related genes. Notably, MSC-EVs and the NUAK1 inhibitor HTH both significantly downregulate NUAK1, inhibit YAP nuclear translocation, block this pathway, and alleviate renal fibrosis.

To clarify the molecular mechanism underlying MSC-EV-mediated NUAK1 downregulation, single-cell transcriptomic data analysis showed that MSC-EVs restore ubiquitin ligase activity in DKD. In recent decades, E3 ubiquitin ligase NEDD4 has received widespread attention owing to its crucial role in regulating various physiological processes and human diseases [27,28]. An increasing body of evidence has confirmed that NEDD4 exerts protective effects against tissue damage, such as hepatic and cardiac ischemia/reperfusion injury [[29], [30], [31]]. Through the UbiBrowser database, we found that NUAK1 can bind to the E3 ubiquitin ligase Nedd4. Molecular docking simulated the protein-protein interaction binding sites of the Nedd4/NUAK1 complex, and We further performed a cell-based ubiquitination assay using wild-type and catalytically inactive Nedd4 constructs to evaluate whether Nedd4 promotes NUAK1 ubiquitination in an E3 ligase activity-dependent manner. Using LC-MS/MS and Western blot, we revealed that MSC-EVs carry the E3 ubiquitin ligase Nedd4 to promote NUAK1 ubiquitination and degradation, a key mechanism for the anti-fibrotic effects of MSC-EVs. To confirm Nedd4 as a key molecule in the anti-fibrotic action of MSC-EVs, we developed proximal tubular epithelial-specific Nedd4-deficient mice (Nedd4^CKO^). The markedly reduced therapeutic effects of MSC-EVs in these mice indicate that Nedd4 is essential for MSC-EV-mediated NUAK1 ubiquitination and degradation, and is a critical target protein against DKD fibrosis.

Owing to their innate low immunogenicity, excellent biocompatibility, and efficient drug-loading capacity, EVs are regarded as a promising novel drug delivery vehicle [32,33]. To further enhance therapeutic efficiency, researchers have subjected MSC-EVs to various engineering modifications [34]. These modifications involve functionalizing their surface and encapsulating specific therapeutic molecules within the vesicles [19]. The aim is to improve their targeting of specific tissues and boost drug delivery efficiency. In addition, using an external magnetic field for guidance is an effective targeting delivery strategy. By imparting superparamagnetic properties to EVs, their magnetic responsiveness can be utilized. When an external magnetic field is applied in vitro, EVs can be directed to migrate towards and accumulate at specific sites of tissue damage. This approach significantly enhances the precision of targeted EV delivery [20,35]. To enhance targeting and efficacy, we constructed a GFP-labeled Nedd4 lentiviral vector to transfect MSCs. Then, using the EXODUS automatic exosome isolation system, we separated MSC-EVs-Nedd4. Transferrin-modified SPION were combined with transferrin receptors on the MSC-EVs-Nedd4 membrane to create dual-targeted engineered EVs (SPION-EVs-Nedd4). Here, the 'dual-targeting' strategy leverages two distinct mechanisms: (1) the inherent propensity of MSC-EVs to accumulate at sites of inflammation and injury (biological targeting), and (2) the precise guidance provided by SPIONs under an external magnetic field (physical targeting). Our study showed that in a DKD model, SPION-EVs-Nedd4 has stronger anti-fibrotic ability and better kidney targeting. These findings deepen our understanding of how MSC-EVs work in DKD interstitial fibrosis and offer key theoretical and experimental support for developing novel MSC-EV-based therapies.

Emerging nanotherapeutic agents have shown great promise in treating renal fibrosis. For instance, Mulberry-derived carbon dots (M-CDs) have been demonstrated to attenuate kidney fibrosis by directly inhibiting PI3K [36]. While such small-molecule-based nanotherapies are highly effective, our SPION-EVs-Nedd4 system provides a complementary strategy by utilizing targeted protein degradation. By delivering the E3 ligase Nedd4, our platform facilitates the ubiquitination of NUAK1, offering a more sustained therapeutic effect than transient signaling inhibition. Furthermore, when compared to synthetic nanoparticles designed to deliver Nedd4 mRNA or NUAK1 siRNA, this engineered EV platform exhibits distinct advantages. Unlike synthetic vectors that may trigger immune clearance, MSC-EVs possess innate biological homing capabilities and low immunogenicity. Direct delivery of the functional Nedd4 protein also circumvents the inherent limitations of nucleic acid delivery, such as the need for cytoplasmic translation (for mRNA) or the risk of off-target effects (for siRNA), thereby providing a more direct and efficient intervention for DKD-associated fibrosis.

Despite the promising anti-fibrotic effects of SPION-EVs-Nedd4, several limitations should be acknowledged. First, while our results demonstrate that Nedd4-mediated NUAK1 degradation is a key mechanism, further studies utilizing conditional NUAK1 knockout models or long-term pharmacological inhibition alone would be beneficial to more comprehensively establish the causal relationship and compare the potency of engineered EVs versus standalone therapies. Second, although we observed significant improvement in histological markers and blood biochemical parameters (BUN and CREA), future research should incorporate more extensive functional indicators, such as 24-h urinary protein levels and measured glomerular filtration rate (mGFR), over longer observation periods to fully validate the translational potential and long-term durability of this platform in clinical settings.

In conclusion, this study offers a novel perspective and potential therapeutic targets for treating DKD interstitial fibrosis. Given their ability to modulate the immune microenvironment, interfere with signaling pathways, and mediate protein transport, MSC-EVs show great potential in suppressing DKD interstitial fibrosis. Future research could further optimize the preparation and modification of MSC-EVs to enhance their therapeutic effects and safety, thereby promoting their clinical translation.

## 4 Materials and methods

### 4.1 Ethics statement on human samples and animal models

Written informed consent was obtained from healthy donors for the collection of discarded human umbilical cord tissue, and kidney biopsy specimens from patients with chronic kidney disease were utilized, which was conducted in accordance with a protocol approved by the Institutional Review Board (IRB: 2022264) of the Affiliated Hospital of Jiangsu University. This study adhered to the ethical principles outlined in the World Medical Association's Declaration of Helsinki. All animal experimental protocols were approved by the Animal Care and Use Committee of Jiangsu University (approval number: 2020280) and carried out in compliance with the relevant guidelines and regulations for work with live vertebrates.

### 4.2 Animals and treatments

Male Sprague-Dawley rats (8 weeks old, 150 g) were purchased from the Jiangsu Experimental Animal Center and housed in a specific pathogen-free environment at the Animal Center of Jiangsu University, with a 12 h light/12 h dark cycle. To establish the DKD rat model, 8-week-old male SD rats were first fed a high-fat diet (HFD) for 4 weeks to induce insulin resistance. Subsequently, a single dose of streptozotocin (STZ, 35 mg/kg) was injected via the tail vein. Following STZ injection, the HFD was continued for another 24 weeks to ensure the stable development of DKD. The db/db mice were also used as DKD model. All animal experiments complied with the US NIH Guide for the Care and Use of Laboratory Animals and were approved by the Ethics Committee of Jiangsu University (2020280, Jiangsu, China). Regarding therapeutic interventions, MSC-EVs or SPION-EVs-Nedd4 were quantified by total vesicle protein using the BCA assay. Each rat received 2 mg EV protein per injection via tail vein once every 7 days, corresponding to an approximately ∼10 mg/kg dose range depending on body weight during treatment. Unless otherwise indicated, EV doses reported in this study refer to the amount of total EV protein per injection. Treatment started at week 8 (post-STZ injection), once persistent albuminuria and renal dysfunction were confirmed, and continued every 7 days for 16 weeks, ending at week 24.

Renal injury was induced in Sprague-Dawley rats via ureteral obstruction (UUO). Rats were anesthetized with 2% isoflurane, and the left ureter was exposed through a lateral incision and ligated with two surgical sutures. Rats were divided into three groups: control, Unilateral ureteral obstruction (UUO) model, and MSC-EVs treatment. The UUO group underwent ureteral ligation for 2 weeks, after which MSC-EVs (2 mg EV protein per rat per injection, quantified by BCA assay) were administered intravenously every 3 days. Renal function was assessed by analyzing serum and blood samples collected after 2 weeks of treatment.

To construction of the Nedd4^CKO^ mouse model, offspring were obtained by mating Nedd4^flox/flox^ mice (with loxP sites on the flanks of the Nedd4 gene exon) and SLC5a12-CRE mice (expressing Cre recombinase in the Slc5a12 promoter region of renal tubular epithelial cells). The offspring were mated with Nedd4^flox/flox^ mice to form homozygous deletion mice expressing the Nedd4 gene in Slc5a12 cells, which were renal tubular epithelial cell-specific Nedd4 gene knockout mice (Nedd4^CKO^). Renal tubular epithelial cell-specific Nedd4 gene knockout mice and wild-type mice were used to induce DKD models with high-fat diet combined with streptozotocin, and MSC-EVs were injected into the tail vein for intervention.

### 4.3 Cell culture

Umbilical cord MSCs were isolated and identified in accordance with the standardized culture process**.** The rat renal tubular cell line NRK-52E was purchased from the National Certified Cell Bank. NRK-52E cells were maintained in DMEM (GIBCO, USA) containing 10% fetal bovine serum, 100 U/mL penicillin, and 100 mg/mL streptomycin in a 5% CO_2_ incubator at 37 °C. Human mesenchymal stem cells were isolated from umbilical cords as previously described. Briefly, umbilical cords from full-term neonates were washed with PBS, and arteries and veins were cut. The tissue was cut into 2-mm pieces, placed in 6-well plates at 5-mm intervals, and cultured in basic DMEM (GIBCO, USA) with 10% fetal bovine serum, 100 U/mL penicillin, and 100 mg/mL streptomycin in a 5% CO_2_ incubator at 37 °C. The culture medium was refreshed every 3 days until mesenchymal stem cell purity reached 85%.

### 4.4 Histological analysis

The kidney samples were fixed in a 10% formalin solution, embedded in paraffin, sectioned at 5 μm, and stained with hematoxylin-eosin (HE), Sirius red, and Masson's trichrome. For histological assessment, the corticomedullary junction regions of kidney sections were analyzed. HE staining evaluated tubular injury, while Sirius red and Masson's trichrome staining assessed collagen deposition in the interstitium. ImageJ quantified the positive areas of Sirius red (red) and Masson's trichrome (blue) staining.

### 4.5 RNA extraction and RT-qPCR

Total RNA was extracted from cells and tissues using the Trizol reagent (Invitrogen) according to the manufacturer's instructions. The RNA concentration was precisely quantified to provide optimal conditions for downstream analysis. The extracted RNA was reverse-transcribed into cDNA using the HiScript III 1st-Strand cDNA Synthesis Kit (gDNA). This step transformed the RNA into a form suitable for quantitative gene expression analysis. AceQ qPCR SYBR Green Master Mix was used for quantitative analysis of target gene expression, with quantification performed using the 2^^−ΔΔCt^ method. β-actin served as an endogenous reference control to normalize gene expression levels.

### 4.6 Western blotting analysis

For western blotting, renal tubular cells and tissues were lysed in lysis buffer. The protein concentration was determined by BCA assay, and the lysates were denatured at 100 °C for 5 min. Proteins were separated by SDS-PAGE, transferred to PVDF membranes, and blocked with 5% BSA. The membranes were incubated with primary rabbit polyclonal antibodies against CD9, CD81, TSG101, Alix, Calnexin (1:500, CST, USA), NUAK1 (1:500, CST, USA), YAP (1:500, CST, USA), CollagenI (1:1000, CST, USA), α-SMA (1:500, BioWorld, USA), Nedd4 (1:500, CST, USA), and TfR (1:500, CST, USA) overnight at 4 °C with constant shaking. After washing five times, the membranes were incubated with HRP-conjugated secondary antibodies for 1 h at room temperature. Protein bands were visualized by western blotting, and band intensities were quantified using ImageJ software. Relative protein expression levels were normalized to β-actin.

### 4.7 Co-immunoprecipitation (Co-IP) assay

Protein A/G magnetic beads (ThermoFisher) were washed four times with 0.5% PBS-Triton X-100. Then, antibody solution was added, and the beads and antibodies were incubated on a rotating mixer at 25 °C for 1 h. The antibody-coupled beads were washed again four times. Next, the prepared protein supernatant was added, and the mixture was incubated on a rotating mixer at 4 °C for 2 h to couple the antibodies and proteins to the beads. After another wash, 1 × SDS-PAGE loading buffer was added, the samples were heated to denature the proteins, and protein content was measured by western blotting.

### 4.8 Single-cell RNA sequencing and data analysis

Renal tissues were collected from three experimental groups, including Control, DKD, and DKD treated with MSC-derived extracellular vesicles (MSC-EVs) (n = 3 sample per group for scRNA-seq). The species used in this study was rat. Single-cell suspensions were prepared from freshly isolated kidney tissues and subjected to single-cell RNA sequencing.

Single-cell libraries were constructed using the 10 × Genomics Chromium Single Cell 3′ v3 platform, following the manufacturer's protocol. Sequencing was performed to generate sufficient depth for transcriptome profiling. Across the three samples, a total of 8264 (Control), 8825 (DKD), and 10,335 (DKD + MSC-EVs) high-quality cells were obtained, with a mean sequencing depth ranging from approximately 51,000 to 72,000 reads per cell and median detected genes per cell ranging from 831 to 1436.

Raw sequencing data were processed using the Cell Ranger pipeline (v3.1.0) for demultiplexing, alignment to the rat reference genome, and generation of the gene expression matrix.

Quality control and downstream analyses were performed using the Seurat R package. Low-quality cells were filtered based on multiple metrics, including the number of detected genes, total UMI counts, and the proportion of mitochondrial gene expression. Cells with extremely low or high gene/UMI counts (outside mean ± 2 standard deviations) were excluded to remove potential dead cells and multiplets. Additionally, cells with elevated mitochondrial transcript proportions were filtered out based on distribution inspection (Supplementary Fig. 6).

Doublets and multiplets were minimized through stringent quality filtering based on UMI/gene distributions, as commonly applied in droplet-based single-cell sequencing workflows. No explicit batch correction was applied, as samples were processed under comparable experimental conditions.

After normalization using Seurat's library size normalization method, highly variable genes were identified. Principal component analysis (PCA) was performed for dimensionality reduction, followed by graph-based clustering. Cell clusters were visualized using t-distributed stochastic neighbor embedding (t-SNE).

Differentially expressed genes (DEGs) were identified using the Seurat statistical framework based on likelihood ratio tests. Genes with |log2 fold change| > 0.58 and adjusted p-value <0.05 were considered significantly differentially expressed.

Cell-type annotation was performed using a combined strategy. First, automated annotation was conducted using the SingleR algorithm, which assigns cell identity based on reference transcriptomic datasets. Subsequently, annotations were manually curated and validated using canonical marker genes. Marker gene expression patterns across clusters are presented in Supplementary heatmaps (Supplementary Fig. 7), supporting the robustness of cell-type identification.

### 4.9 Immunohistochemistry and immunofluorescence

Paraffin-embedded tissue sections underwent heat fixation, dewaxing, rehydration, antigen retrieval, and further processing. Renal tissue sections were fixed at 60 °C for 2 h, dewaxed in xylene, and rehydrated in ethanol. Antigen retrieval was performed with citrate buffer under high pressure for 30 min. Sections were incubated with 3% H_2_O_2_ in the dark for 10 min, blocked with 10% BSA for 1 h, and stained overnight at 4 °C with α-SMA, NUAK1, and YAP antibodies, followed by three PBS washes. HRP-conjugated goat anti-mouse/rabbit IgG was applied for 30 min, and after three PBST washes, DAB substrate was added for color development. Sections were counterstained with hematoxylin, dehydrated in ethanol and xylene, and imaged using a Leica fluorescence microscope.

Renal tissue sections, after dewaxing and rehydration, were stained overnight at 4 °C with antibodies against α-SMA, Smad2/3, CD68, iNOS, CD206, VEGF, TGF-β1, NUAK1, YAP, Nedd4, and Slc5a12, followed by three PBS washes. DAPI staining was performed for imaging with a confocal laser-scanning microscope (GE, USA). NRK-52E cells were washed twice with cold PBS, fixed in 4% PFA for 10 min, blocked with 10% BSA at room temperature for 1 h, and stained overnight at 4 °C with antibodies against NUAK1, YAP, Nedd4, α-SMA. After three cold PBS washes, cells were stained with DAPI for confocal laser-scanning microscopy (GE, USA).

### 4.10 Macrophage and renal tubular epithelial cell co-culture

First, macrophages were cultured in a high glucose environment for 48 h. Then, primary renal tubular epithelial cells (20,000 cells) and macrophages (15,000 cells) from individual DKD rats (n = 5) were seeded into Gibco Dulbecco's Modified Eagle Medium. All growth factor supplements were removed, and the cells were cultured in the basic medium for 72 h.

### 4.11 Molecular docking

The crystal structure of Nedd4 was obtained from the RCSB PDB database (5OAQ). The full-length structure of NUAK1 was downloaded from the AlphaFold Protein Structure Database and used for molecular docking analysis. Molecular docking was performed using the ZDOCK server, and the docking results were visualized with PyMOL v2.5.0.

### 4.12 Protein stability assays and ubiquitination assays

Cells were cultured according to the aforementioned method. When the cell density reached 60% to 70%, Cycloheximide (CHX) (100 μg/mL) was added for 8, 10, and 12 h, respectively. Cells were then collected, and Western blot (WB) analysis was performed to detect NUAK1 protein stability. The Flag-ubiquitin plasmid was used to overexpress ubiquitin. Subsequently, cells were treated with the proteasome inhibitor MG132 (20 μmol/L) for 6 h before harvest. Co-immunoprecipitation (Co-IP) was conducted using an anti-Flag-ubiquitin antibody, followed by WB detection of ubiquitin.

### 4.13 Extraction and purification of SPION-EVs-Nedd4

The lentiviral vector encoding mouse Nedd4 was purchased from Genepharma (Suzhou, China). Mesenchymal stem cells were transfected with Nedd4-encoding lentivirus. When transfection efficiency reached 80%, Nedd4 expression post-transfection was analyzed and measured via immunofluorescence and imaging flow cytometry. EVs in the culture medium and FBS were depleted through ultracentrifugation. After 48 h, the culture medium was replaced and collected. MSC-EVs were isolated and purified via ultracentrifugation. Briefly, the medium was centrifuged at 800 g for 10 min, 2000 g for 10 min, 10,000 g for 30 min, and 100,000 g for 70 min at 4 °C to obtain MSC-EVs-Nedd4. Their morphology was examined by TEM.NanoSight analysis was used to measure zeta potential and nanoparticle size. Immunofluorescence was used to detect Nedd4/NUAK1 expression in NRK-52E cells treated with MSC-EVs-Nedd4. Furthermore, superparamagnetic iron oxide nanoparticles (SPIONs) modified with transferrin were co-incubated with MSC-EVs-Nedd4 at 37 °C for 4 h to form SPION-EVs-Nedd4.

### 4.14 Biological characterization of SPION-EVs-Nedd4

The prepared MSC-EVs and SPION-EVs samples were applied to 300-mesh copper grids and incubated for 3 min. Then, 20 μL of 1% uranyl acetate was added to the grids and incubated for 1 min, followed by washing and drying for TEM observation. Images were obtained using a transmission electron microscope. Western blotting was performed to assess the expression of EV-specific markers CD9 and TSG101, as well as Nedd4 and TfR. Organ distribution of SPION-EVs-Nedd4 was evaluated with a small-animal in vivo imaging system.

### 4.15 Biodistribution analysis

To evaluate the biodistribution of engineered EVs, MSC-EVs and SPION-EVs-Nedd4 were labeled with the near-infrared fluorescent dye CM-DiR. Briefly, 200 μg of EVs were incubated with 1 μM CM-DiR at 37 °C for 30 min, followed by ultracentrifugation at 100,000 g for 1 h to remove excess dye. For the in vivo study, DKD rats received a single tail-vein injection of CM-DiR-labeled EVs at approximately 10 mg/kg (based on total EV protein quantified by BCA assay). At 24 h post-injection, the rats were euthanized, and major organs (heart, liver, spleen, lung, and kidney) were harvested. The fluorescence intensity in each organ was captured using an IVIS Lumina imaging system (Xenogen, USA) with an excitation/emission wavelength of 748/780 nm. The radiant efficiency [(p/sec/cm^2^/sr)/(μW/cm^2^)] was quantified using Living Image software (v4.5). To ensure accuracy, the background fluorescence of organs from saline-injected rats was subtracted.

### 4.16 Statistical analysis

All experiments were performed at least three times for each group, and the statistical analyses were performed using GraphPad Prism Software (version 7). The results were presented as mean values ± standard deviation. For all bar graphs, n represents the number of independent biological replicates or individual animals. One-way analysis of variance (ANOVA) and two-way ANOVA for multiple groups and Student's *t*-test for two groups were applied for statistical analysis. Survival time was analyzed using the Kaplan-Meier method and log-rank test. A *P* value < 0.05 indicated statistical significance.

## Ethics approval and consent to participate

All animal experiments were performed in strict accordance with the National Institutes of Health Guide for the Care and Use of Laboratory Animals guidelines and were approved by the Ethics Committee of Jiangsu University (2020280, Jiangsu, China).

## CRediT authorship contribution statement

**Cheng Ji:** Writing – original draft, Writing – review & editing. **Bei Li:** Data curation, Methodology, Software. **Jiahui Zhang:** Data curation, Methodology, Software. **Linru Shi:** Data curation, Methodology. **Leiyi Zhang:** Data curation, Software. **Hui Shi:** Project administration, Supervision. **Xu Zhang:** Project administration, Supervision. **Wenrong Xu:** Supervision. **Lixia Yu:** Supervision. **Qifeng Liu:** Project administration, Resources, Supervision, Writing – review & editing. **Hui Qian:** Project administration, Resources, Supervision, Writing – review & editing.

## Declaration of competing interest

The authors declare that they have no known competing financial interests or personal relationships that could have appeared to influence the work reported in this paper.

## Data Availability

Data will be made available on request.

## References

[1] Abbad L., Esteve E., Chatziantoniou C. (2025). Advances and challenges in kidney fibrosis therapeutics. Nat. Rev. Nephrol..

[2] Tian S., Zhou S., Wu W., Lin Y., Wang T., Sun H., A-Ni-Wan A.-S.-J., Li Y., Wang C., Li X., Yu P., Zhao Y. (2025). GLP-1 receptor agonists alleviate diabetic Kidney injury via β-Klotho-Mediated ferroptosis inhibition. Adv. Sci. (Weinh.).

[3] Francis A., Harhay M.N., Ong A.C.M., Tummalapalli S.L., Ortiz A., Fogo A.B., Fliser D., Roy-Chaudhury P., Fontana M., Nangaku M., Wanner C., Malik C., Hradsky A., Adu D., Bavanandan S., Cusumano A., Sola L., Ulasi I., Jha V., American Society of Nephrology, European Renal Association, International Society of Nephrology (2024). Chronic kidney disease and the global public health agenda: an international consensus. Nat. Rev. Nephrol..

[4] Xu F., Jiang H., Li X., Pan J., Li H., Wang L., Zhang P., Chen J., Qiu S., Xie Y., Li Y., Zhang D., Dong Z. (2024). Discovery of PRDM16-Mediated TRPA1 induction as the mechanism for low tubulo-interstitial fibrosis in diabetic kidney disease. Adv. Sci. (Weinh.).

[5] Lamas S., Ruiz-Ortega M. (2025). Insights into the mechanisms of fibrosis and progressive kidney injury. Nat. Rev. Nephrol..

[6] Long T., Lu Y., Ma Y., Song Y., Yi X., Chen X., Zhou M., Ma J., Chen J., Liu Z., Zhu F., Hu Z., Zhou Z., Li C., Hou F.F., Zhang L., Chen Y., Nie J. (2025). Condensation of cellular prion protein promotes renal fibrosis through the TBK1-IRF3 signaling axis. Sci. Transl. Med..

[7] Wu S., Chu X., Lv G., Gao J., Huang Y., Li H., Jiang X., Liu Y., Zhang J., Fang X., Yao Z., Bu W. (2025). Mesenchymal stem cells with polydopamine-coated NaGdF4 nanoparticles with Ca2+ chelation ability for ischemic stroke therapy. Adv. Mater..

[8] Chen Z., Zou Y., Sun H., He Y., Ye K., Li Y., Qiu L., Mai Y., Chen X., Mao Z., Yi C., Wang W. (2024). Engineered enucleated mesenchymal stem cells regulating immune microenvironment and promoting wound healing. Adv. Mater..

[9] Chang W., Tian B., Qin Q., Li D., Zhang Y., Zhou C., Wu B., Zhang M., Shan H., Ni Y., Dong Q., Wang C., Zhou X.-Z., Bai J. (2024). Receptor activator of nuclear factor Kappa-B-Expressing mesenchymal stem cells-derived extracellular vesicles for osteoporosis therapy. ACS Nano.

[10] Dubey S., Chen Z., Jiang Y.J., Talis A., Molotkov A., Ali A., Mintz A., Momen-Heravi F. (2024). Small extracellular vesicles (sEVs)-based gene delivery platform for cell-specific CRISPR/Cas9 genome editing. Theranostics.

[11] Kalluri R., LeBleu V.S. (2020). The biology, function, and biomedical applications of exosomes. Science.

[12] Liao Y., Zhang W., Liu Y., Zhu C., Zou Z. (2024). The role of ubiquitination in health and disease. MedComm.

[13] Maspero E., Cappa A., Weber J., Trifirò P., Amici R., Bruno A., Fagà G., Cecatiello V., Fattori R., Leuzzi B., Taibi V., Meroni G., Pasi M., Romussi A., Sartori L., Villa M., Vultaggio S., Cirò M., Soffientini P., Lombardo L., Dahe S., Bachi A., Varasi M., Rossi M., Pasqualato S., Mercurio C., Polo S. (2025). Structure-based design of potent and selective inhibitors of the HECT ligase NEDD4. Commun. Chem..

[14] Janosev M., Kosek D., Tekel A., Joshi R., Honzejkova K., Pohl P., Obsil T., Obsilova V. (2025). Structural basis of ubiquitin ligase Nedd4-2 autoinhibition and regulation by calcium and 14-3-3 proteins. Nat. Commun..

[15] Zhang Y., Qian H., Wu B., You S., Wu S., Lu S., Wang P., Cao L., Zhang N., Sun Y. (2020). E3 Ubiquitin ligase NEDD4 family-regulatory network in cardiovascular disease. Int. J. Biol. Sci..

[16] Carney E.F. (2022). NUAK1 promotes kidney fibrosis. Nat. Rev. Nephrol..

[17] Zhang T., He X., Caldwell L., Goru S.K., Ulloa Severino L., Tolosa M.F., Misra P.S., McEvoy C.M., Christova T., Liu Y., Atin C., Zhang J., Hu C., Vukosa N., Chen X., Krizova A., Kirpalani A., Gregorieff A., Ni R., Chan K., Gill M.K., Attisano L., Wrana J.L., Yuen D.A. (2022). NUAK1 promotes organ fibrosis via YAP and TGF-β/SMAD signaling. Sci. Transl. Med..

[18] Lee J.-R., Park B.-W., Kim J., Choo Y.W., Kim H.Y., Yoon J.-K., Kim H., Hwang J.-W., Kang M., Kwon S.P., Song S.Y., Ko I.O., Park J.-A., Ban K., Hyeon T., Park H.-J., Kim B.-S. (2020). Nanovesicles derived from iron oxide nanoparticles-incorporated mesenchymal stem cells for cardiac repair. Sci. Adv..

[19] Li B., Qi C., Zhang Y., Shi L., Zhang J., Qian H., Ji C. (2024). Frontier role of extracellular vesicles in kidney disease. J. Nanobiotechnol..

[20] Zhang H., Mao Y., Nie Z., Li Q., Wang M., Cai C., Hao W., Shen X., Gu N., Shen W., Song H. (2024). Iron oxide nanoparticles engineered macrophage-derived exosomes for targeted pathological angiogenesis therapy. ACS Nano.

[21] Zhang J., Ji C., Zhang H., Shi H., Mao F., Qian H., Xu W., Wang D., Pan J., Fang X., Santos H.A., Zhang X. (2022). Engineered neutrophil-derived exosome-like vesicles for targeted cancer therapy. Sci. Adv..

[22] Ji C., Zhang J., Shi L., Shi H., Xu W., Jin J., Qian H. (2024). Engineered extracellular vesicle-encapsulated CHIP as novel nanotherapeutics for treatment of renal fibrosis. npj Regen. Med..

[23] Mizenko R.R., Feaver M., Bozkurt B.T., Lowe N., Nguyen B., Huang K.-W., Wang A., Carney R.P. (2024). A critical systematic review of extracellular vesicle clinical trials. J. Extracell. Vesicles.

[24] Jin C., Xue L., Zhang L., Yu L., Wu P., Qian H. (2025). Engineered nanoparticles for theranostic applications in kidney repair. Adv. Healthcare Mater..

[25] Abedini A., Levinsohn J., Klötzer K.A., Dumoulin B., Ma Z., Frederick J., Dhillon P., Balzer M.S., Shrestha R., Liu H., Vitale S., Bergeson A.M., Devalaraja-Narashimha K., Grandi P., Bhattacharyya T., Hu E., Pullen S.S., Boustany-Kari C.M., Guarnieri P., Karihaloo A., Traum D., Yan H., Coleman K., Palmer M., Sarov-Blat L., Morton L., Hunter C.A., Kaestner K.H., Li M., Susztak K. (2024). Single-cell multi-omic and spatial profiling of human kidneys implicates the fibrotic microenvironment in kidney disease progression. Nat. Genet..

[26] Wang Y.-B., Li T., Wang F.-Y., Yao X., Bai Q.-X., Su H.-W., Liu J., Wang L., Tan R.-Z. (2025). The dual role of cellular senescence in macrophages: unveiling the hidden driver of age-related inflammation in Kidney disease. Int. J. Biol. Sci..

[27] Tang X., Liu X., Sha X., Zhang Y., Zu Y., Fan Q., Hu L., Sun S., Zhang Z., Chen F., Yan C., Chen X., Xu Y., Chen W., Shao Y., Gu J., Pu J., Yu B., Han Y., Xie L., Han Y., Ji Y. (2025). NEDD4-Mediated GSNOR degradation aggravates cardiac hypertrophy and dysfunction. Circ. Res..

[28] Li J., Zhu K., Gu A., Zhang Y., Huang S., Hu R., Hu W., Lei Q.-Y., Wen W. (2023). Feedback regulation of ubiquitination and phase separation of HECT E3 ligases. Proc. Natl. Acad. Sci. U. S. A..

[29] Sun W., Lu H., Cui S., Zhao S., Yu H., Song H., Ruan Q., Zhang Y., Chu Y., Dong S. (2023). NEDD4 ameliorates myocardial reperfusion injury by preventing macrophages pyroptosis. Cell Commun. Signal..

[30] Zhu Y., Lei L., Wang X., Chen L., Li W., Li J., Zhao C., Du X., Song Y., Gao W., Liu G., Li X. (2023). The E3 ubiquitin ligase NEDD4-1 protects against acetaminophen-induced liver injury by targeting VDAC1 for degradation. Acta Pharm. Sin. B.

[31] Li Q., Zhang F., Wang H., Tong Y., Fu Y., Wu K., Li J., Wang C., Wang Z., Jia Y., Chen R., Wu Y., Cui R., Wu Y., Qi Y., Qu K., Liu C., Zhang J. (2024). NEDD4 lactylation promotes APAP induced liver injury through Caspase11 dependent non-canonical pyroptosis. Int. J. Biol. Sci..

[32] Chen Z.-Q., Tang T.-T., Tang R.-N., Zhang Y., Zhang Y.-L., Yang H.-B., Song J., Yang Q., Qin S.-F., Chen F., Zhang Y.-X., Wang Y.-J., Wang B., Lv L.-L., Liu B.-C. (2025). A comprehensive evaluation of stability and safety for HEK293F-derived extracellular vesicles as promising drug delivery vehicles. J. Contr. Release.

[33] Kim H.I., Park J., Zhu Y., Wang X., Han Y., Zhang D. (2024). Recent advances in extracellular vesicles for therapeutic cargo delivery. Exp. Mol. Med..

[34] Sun F., Sun Y., Wang X., Zhu J., Chen S., Yu Y., Zhu M., Xu W., Qian H. (2024). Engineered mesenchymal stem cell-derived small extracellular vesicles for diabetic retinopathy therapy through HIF-1α/EZH2/PGC-1α pathway. Bioact. Mater..

[35] Yang L., Patel K.D., Rathnam C., Thangam R., Hou Y., Kang H., Lee K.-B. (2022). Harnessing the therapeutic potential of extracellular vesicles for biomedical applications using multifunctional magnetic nanomaterials. Small.

[36] Liu T., Wu Y., Wang C., Zhou T., Yang R., Zhang X., Yan W., Fan Q., Lu Z. (2025). Mulberry-derived carbon dots attenuate kidney fibrosis through direct inhibition of PI3K. Mater. Today Bio.

