# Supplementary material for: Dual-targeted engineered mesenchymal stem cell-derived extracellular vesicles delivering Nedd4 attenuate renal fibrosis in diabetic kidney disease
Source: Mater Today Bio. 2026 Apr 5;38:103097. doi: 10.1016/j.mtbio.2026.103097 (PMC13091060; doi:10.1016/j.mtbio.2026.103097)
Supplement: Multimedia component 1 [file mmc1.docx]

**Supplementary Information**

**Dual-targeted engineered mesenchymal stem cell-derived extracellular vesicles delivering Nedd4 attenuate renal fibrosis in diabetic kidney disease**

Cheng Ji^a,b*^, Bei Li^b#^, Jiahui Zhang^b#^, Linru Shi^b^, Leiyi Zhang^c^, Hui Shi^d^, Xu Zhang^d^, Wenrong Xu^d^, Lixia Yu^c^, Qifeng Liu^c*^, Hui Qian^c, d*^

^a^ Department of Nephrology, Affiliated Kunshan Hospital of Jiangsu University, 215300, Kunshan, China.

^b^ Jiangsu Key Laboratory of Laboratory Medicine, Department of laboratory Medicine, School of Medicine, Jiangsu University, 212013, Zhenjiang, China.

^c^ Department of Nephrology, Affiliated Kunshan Hospital of Jiangsu University, 215300, Kunshan, China.

^d^ Jiangsu Key Laboratory of Laboratory Medicine, Department of laboratory Medicine, School of Medicine, Jiangsu University, 212013, Zhenjiang, China.

* Correspondence should be addressed to:

Hui Qian, Jiangsu University, 212013, Zhenjiang, China. E-mail: lstmmmlst@163.com

Qifeng Liu, Affiliated Kunshan Hospital of Jiangsu University, 215300, Kunshan, China. E-mail: lqfeng02@163.com


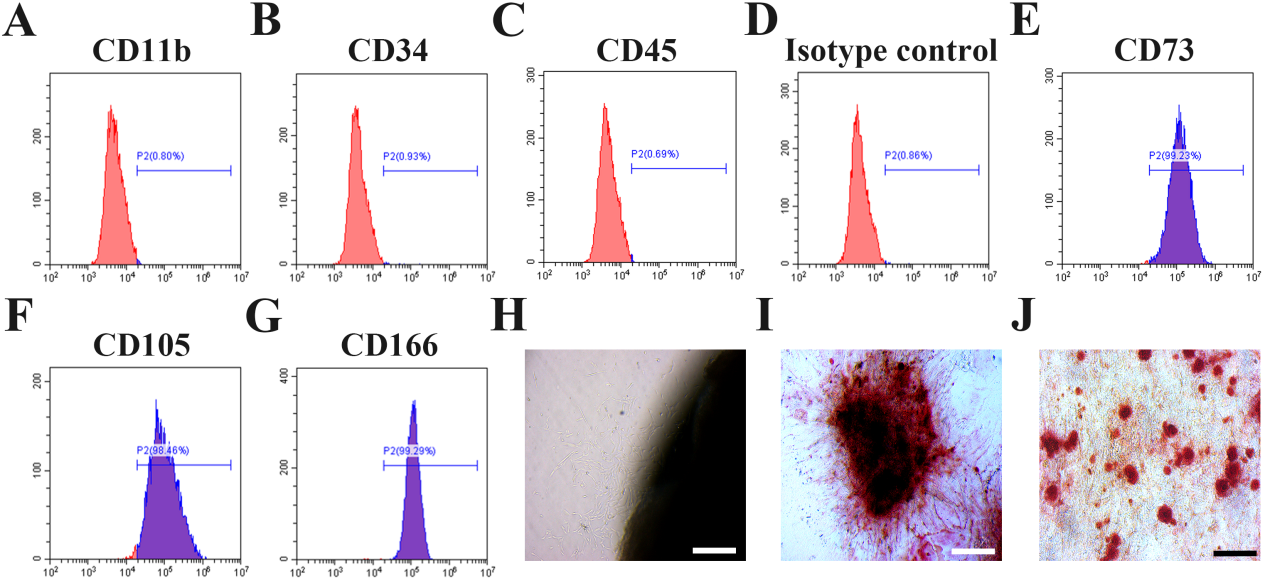


**Supplemental Figure 1.** **Biological characteristics of hucMSCs**. A-G. Immunophenotypes of the human umbilical cord derived MSCs (hucMSCs) were measured by flow cytometry, positive expression of CD73, CD105, and CD166, and negative for CD11b, CD34, and CD45; H. Representative bright-field images of MSCs; I. Alkaline phosphatase (ALP) staining was used to detect the osteogenesis of MSC and MSC-CHIP group (above). Scale bar: 200µm; J. Oil red O staining for detecting lipogenesis in MSC and MSC-CHIP group (below). Scale bar: 500µm.


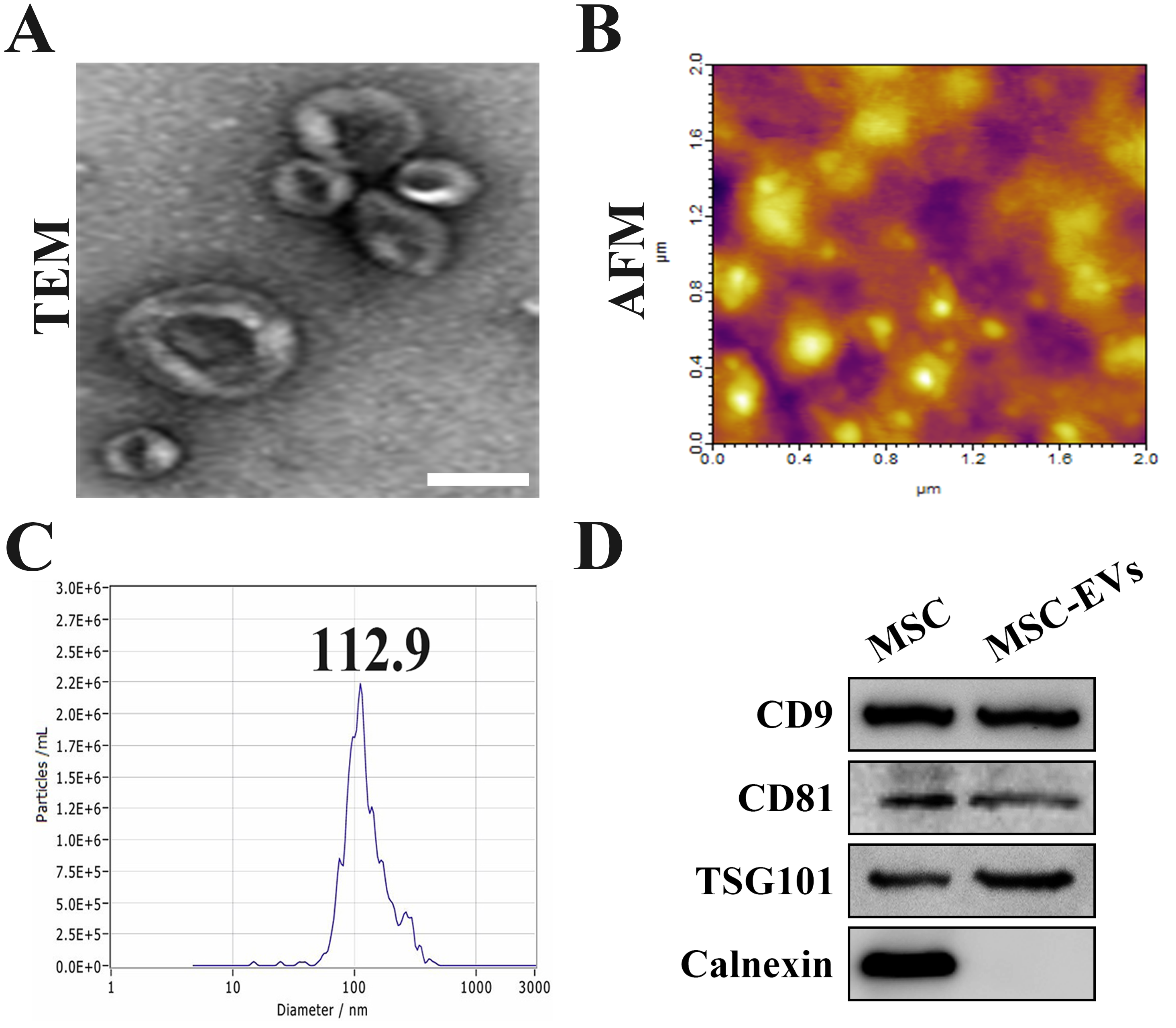


**Supplemental Figure 2. The biological characteristics of MSC-EVs.** A. The TEM morphology of MSC-EVs (scale bar: 100 nm); B. Atomic force microscopy (AFM) detection of MSC-EVs structure; C. Nanoparticle tracking analysis (NTA) measurement of MSC-EVs particle size; D. Western blot detection of protein marker expression in MSC-EVs.


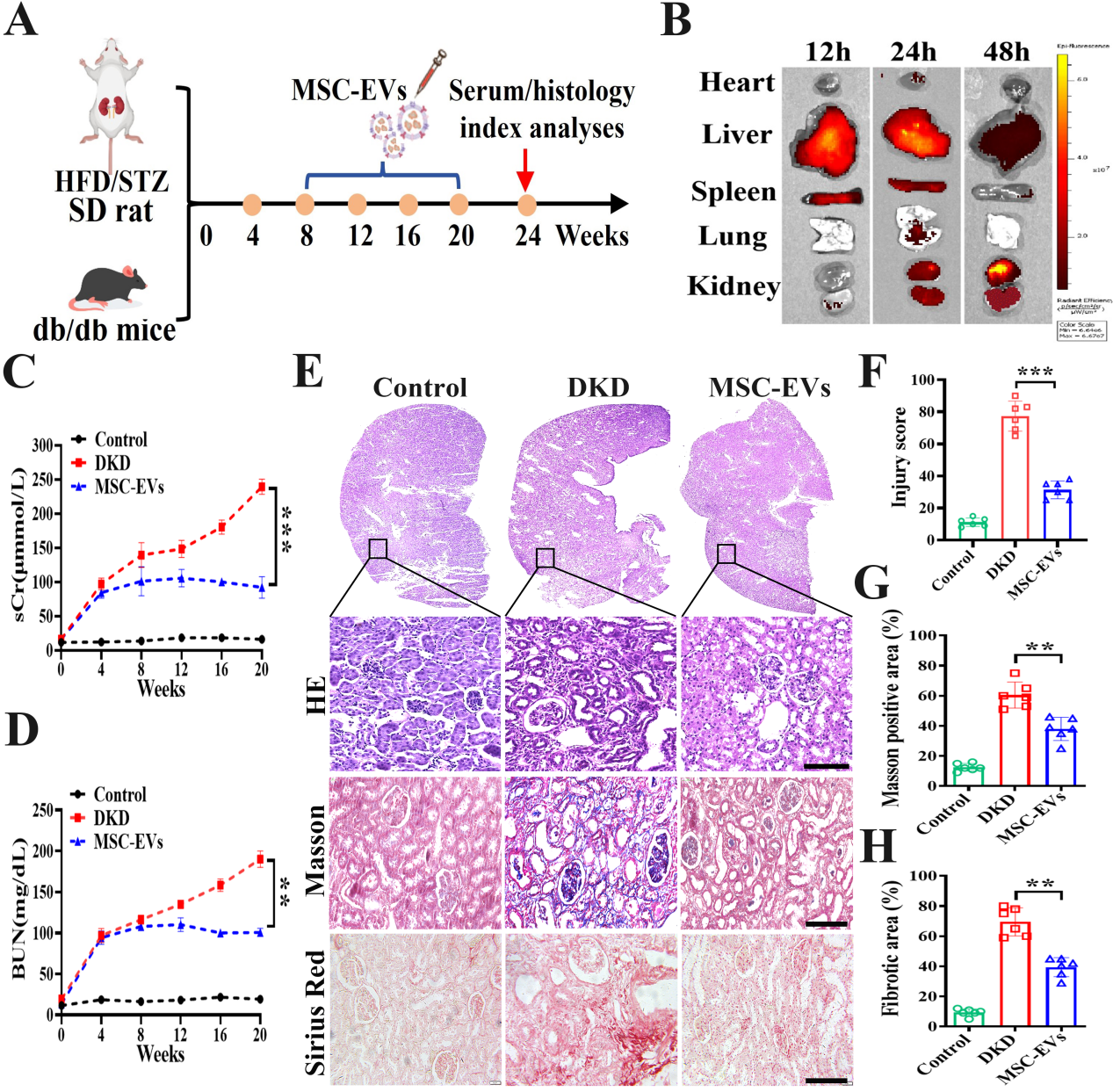


**Supplemental Figure 3. MSC-EVs alleviate interstitial collagen deposition in DKD model.** A. Schematic diagram of MSC-EVs intervention in DKD; B. In vivo distribution of MSC-EVs monitored by a small animal imaging system; C. The changes of serum creatinine and D) urea nitrogen in rats with DKD were detected by biochemical analyzers; E. Representative images of renal H&E staining, Sirius red and Masson's trichrome collagen staining (scale bar: 100 μm); F-H. HE staining was used to evaluate the renal injury score, Sirius red and Masson's trichrome staining were used to assess the rate of renal fibrosis (n = 6). All values are presented as the mean ± SD.***P* < 0.01, ****P* < 0.001

**
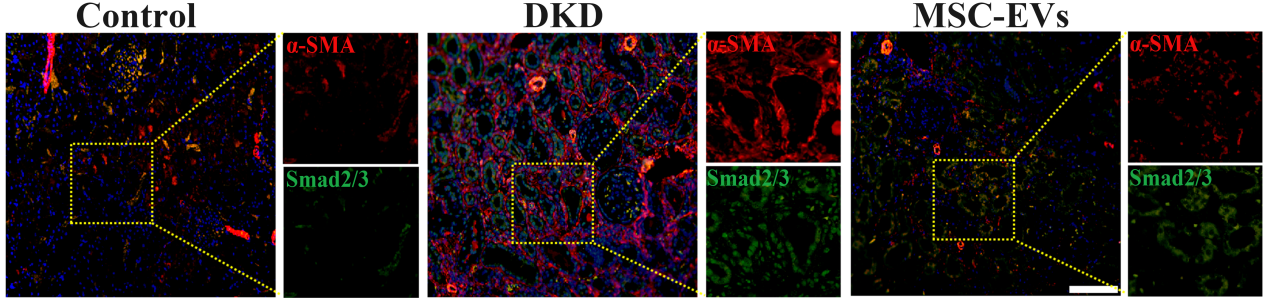
Supplemental Figure 4.** **MSC-EVs inhibit the expression of α-SMA and Smad2/3.** The expressions of α-SMA and Smad2/3 were detected by immunofluorescence (scale bar: 100 μm).


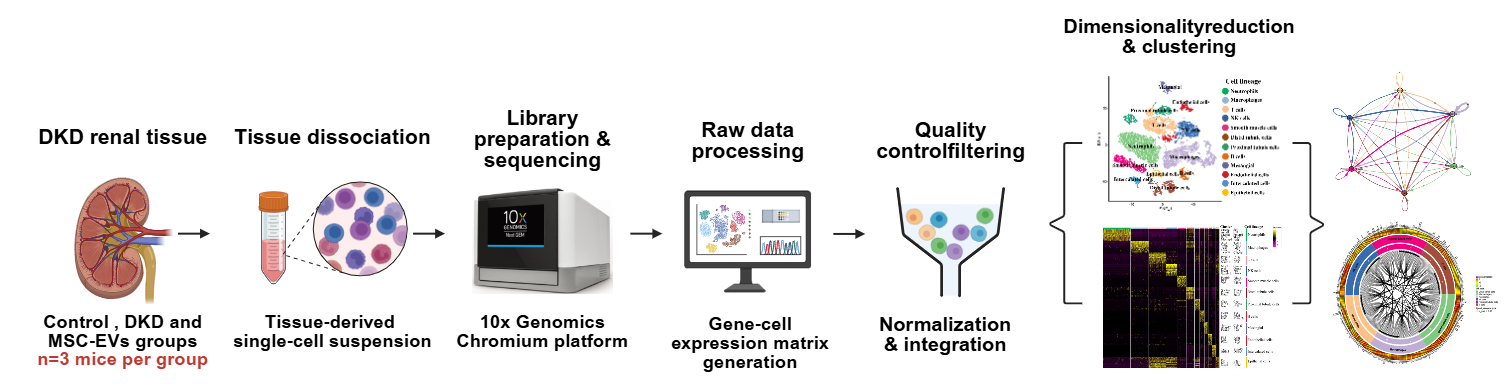


**Supplemental Figure 5. Schematic overview of the single-cell RNA-sequencing workflow used in this study.** Renal tissues were collected from the Control, DKD, and DKD+MSC-EVs groups (n = 3 per group). Fresh kidney tissues were enzymatically dissociated to generate single-cell suspensions, followed by single-cell capture and library construction using the 10× Genomics Chromium Single Cell 3′ v3 platform. After sequencing, raw data were processed using the Cell Ranger pipeline to generate the gene–cell expression matrix. Downstream analyses included quality control and filtering, normalization, dimensionality reduction, clustering, and cell-type annotation, followed by subsequent bioinformatic analyses of intergroup differences.


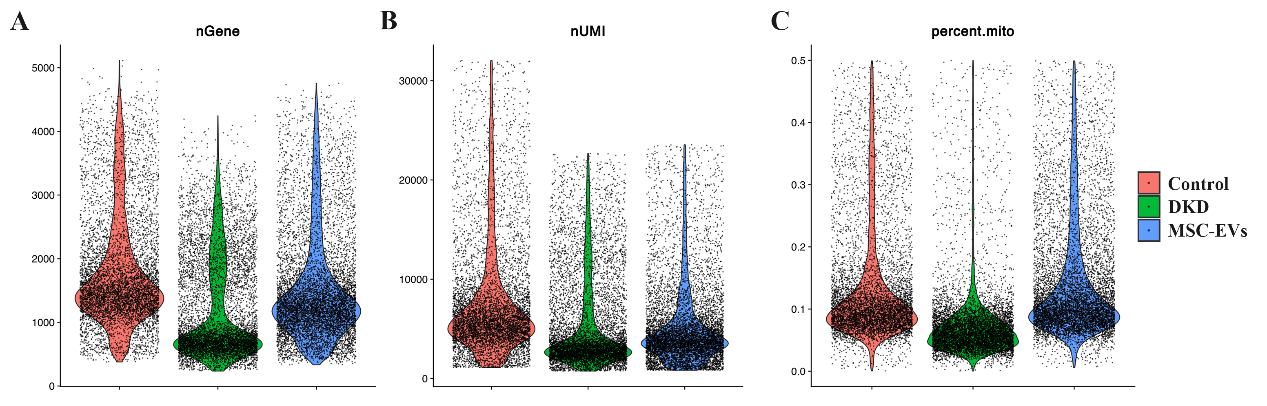


**Supplemental Figure 6.** **Quality control metrics of scRNA-seq data.** A. Distribution of detected gene numbers per cell; B. Distribution of UMI counts per cell; C. Distribution of mitochondrial gene percentages per cell.


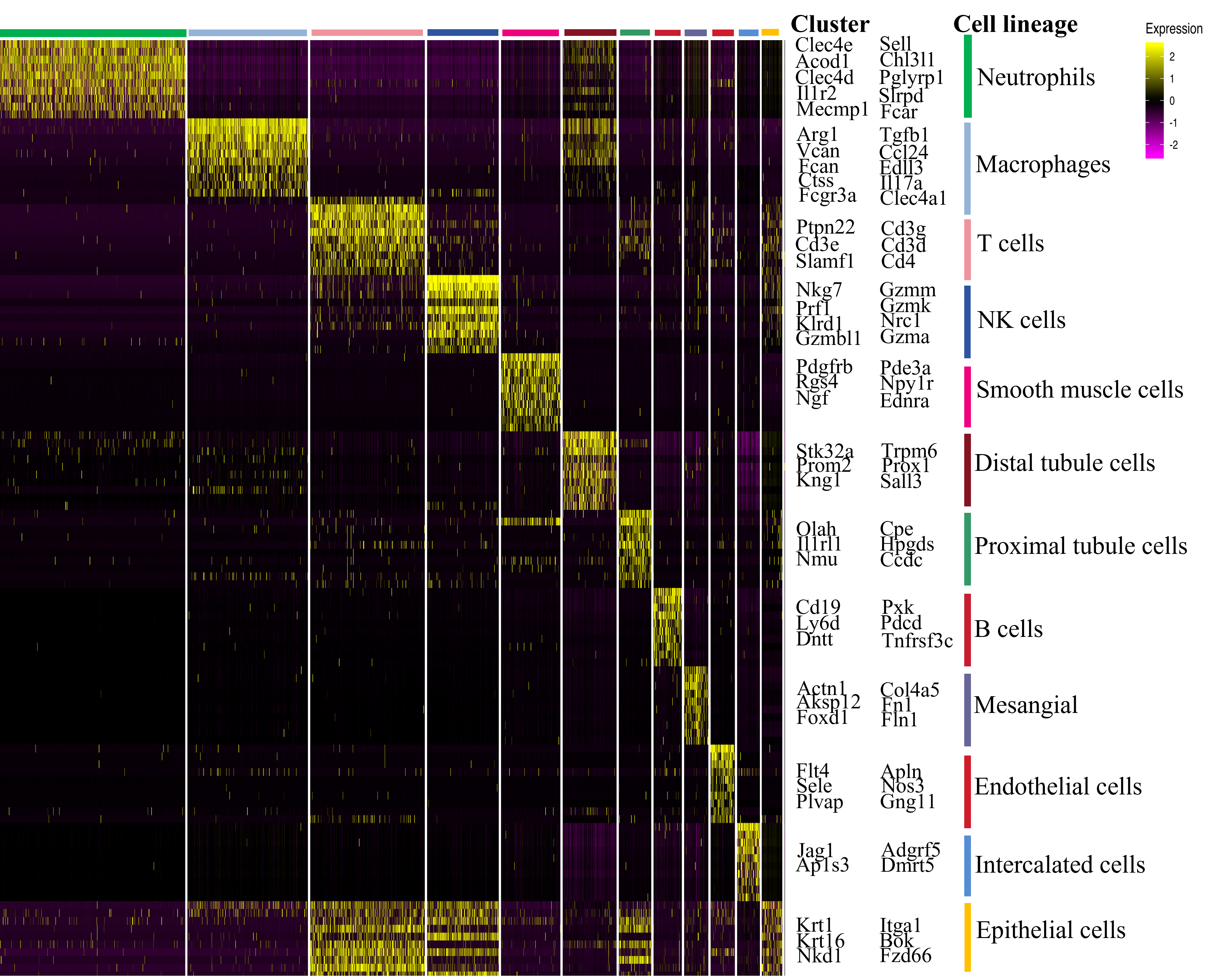


**Supplemental Figure 7.** To support the validity of cell-type annotation, expression patterns of established marker genes for major renal cell populations were visualized using heatmaps.


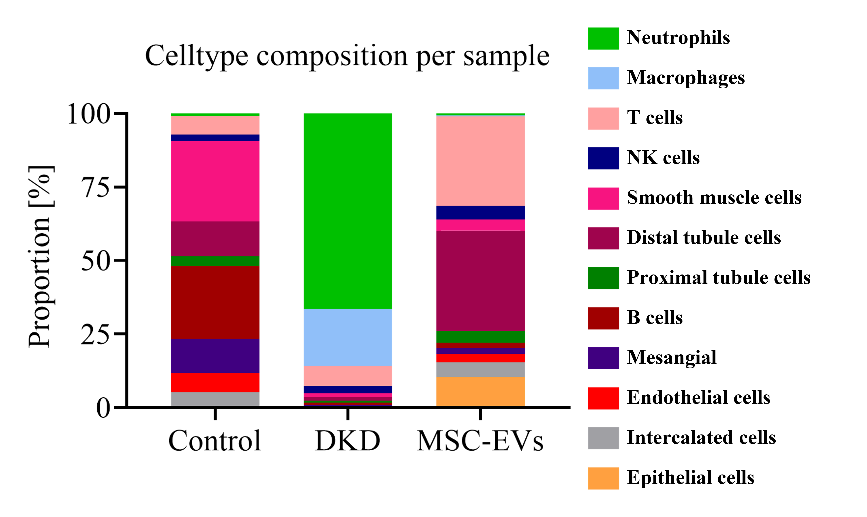


**Supplemental Figure 8.** Global cell-type composition of kidneys across experimental groups. Stacked bar plots showing the relative proportions of major renal cell types in Control, DKD, and DKD + MSC-EVs groups based on single-cell RNA sequencing data. Each bar represents the percentage of total cells assigned to each cell type within a given group. MSC-EVs treatment partially reversed DKD-induced alterations in renal cellular composition, specifically reducing macrophage accumulation.


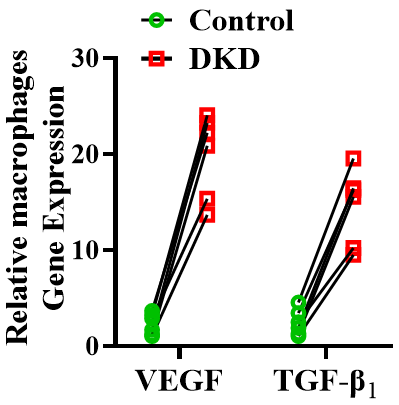


**Supplemental Figure 9.** The expression levels of VEGF and TGF-β_1_ in macrophages of DKD renal tissues were detected by qRT-PCR (n = 6).


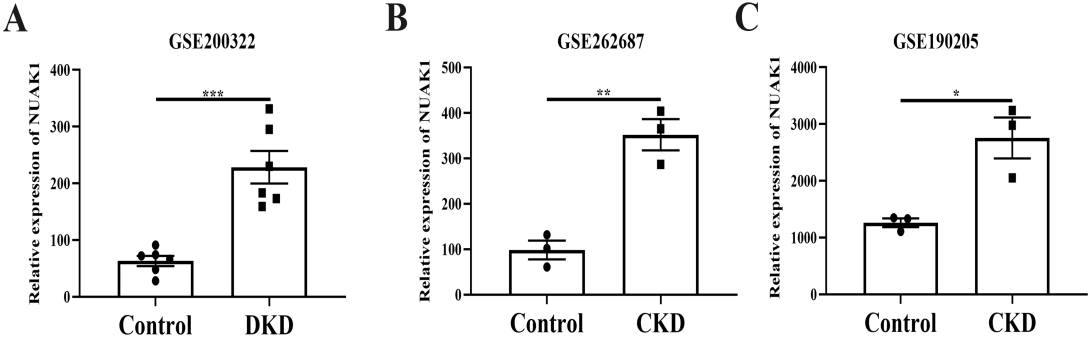


**Supplemental Figure 10.** The mRNA levels of NUAK1 in renal tubules obtained from the GEO database (GSE200322, 262687,190205). All values are presented as the mean ± SD.**P* < 0.05, ***P* < 0.01, ****P* < 0.001.


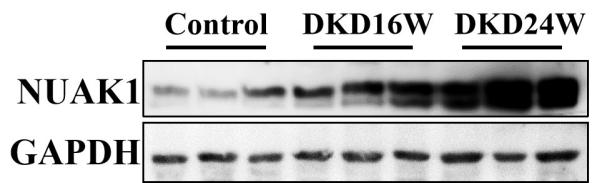


**Supplemental Figure 11.** The expression of NUAK1 in 16 weeks and 24 weeks DKD renal tissue was determined by western blotting.


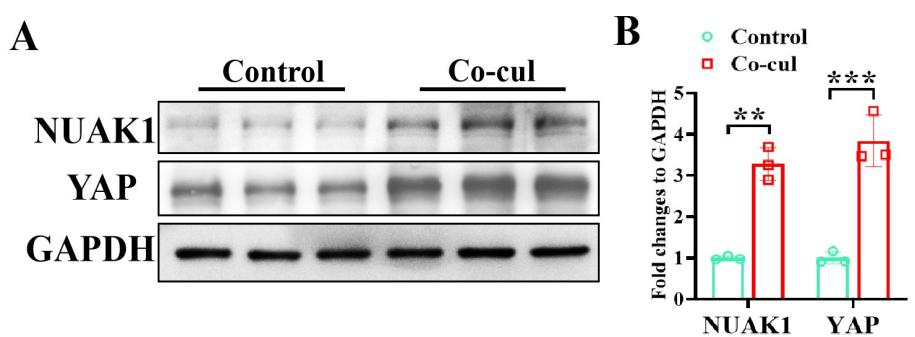


**Supplemental Figure 12.** **Macrophages stimulate the activation of the NUAK1/YAP pathway in renal tubular epithelial cells.** A-B. NUAK1 and YAP expression in NRK-52E cells under macrophage co-culture conditions was determined by western blotting (n=3). All values are presented as the mean ± SD. ***P* < 0.01, ****P* < 0.001.


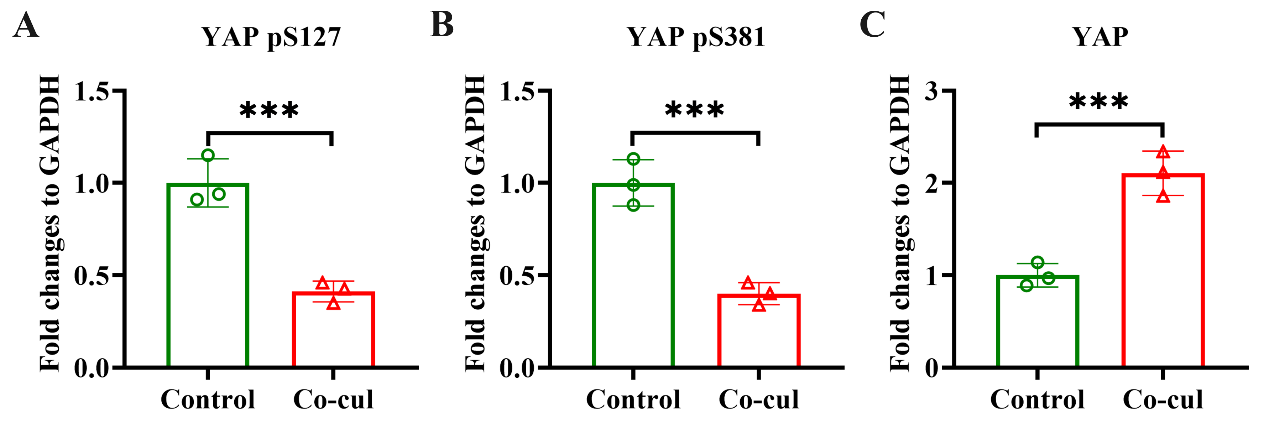


**Supplemental Figure 13.** Quantitative analysis of Western blot bands shown in Figure 2P (n=3). All values are presented as the mean ± SD. ****P* < 0.001.


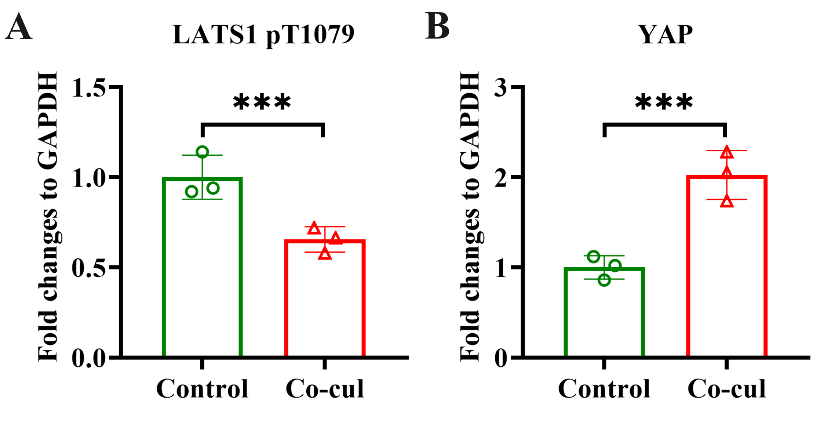


**Supplemental Figure 14.** Quantitative analysis of Western blot bands shown in Figure 2R (n=3). All values are presented as the mean ± SD. ****P* < 0.001.


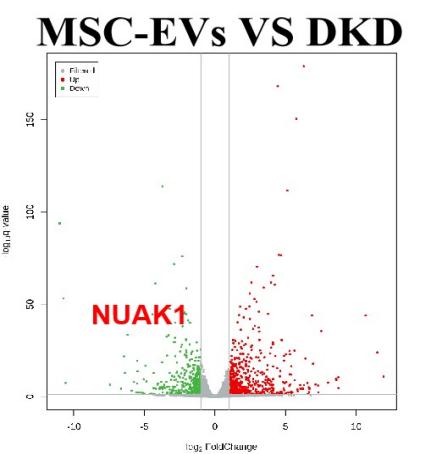


**Supplemental Figure 15.** Top gene expression profile in MSC-EVs and DKD groups renal tissue.


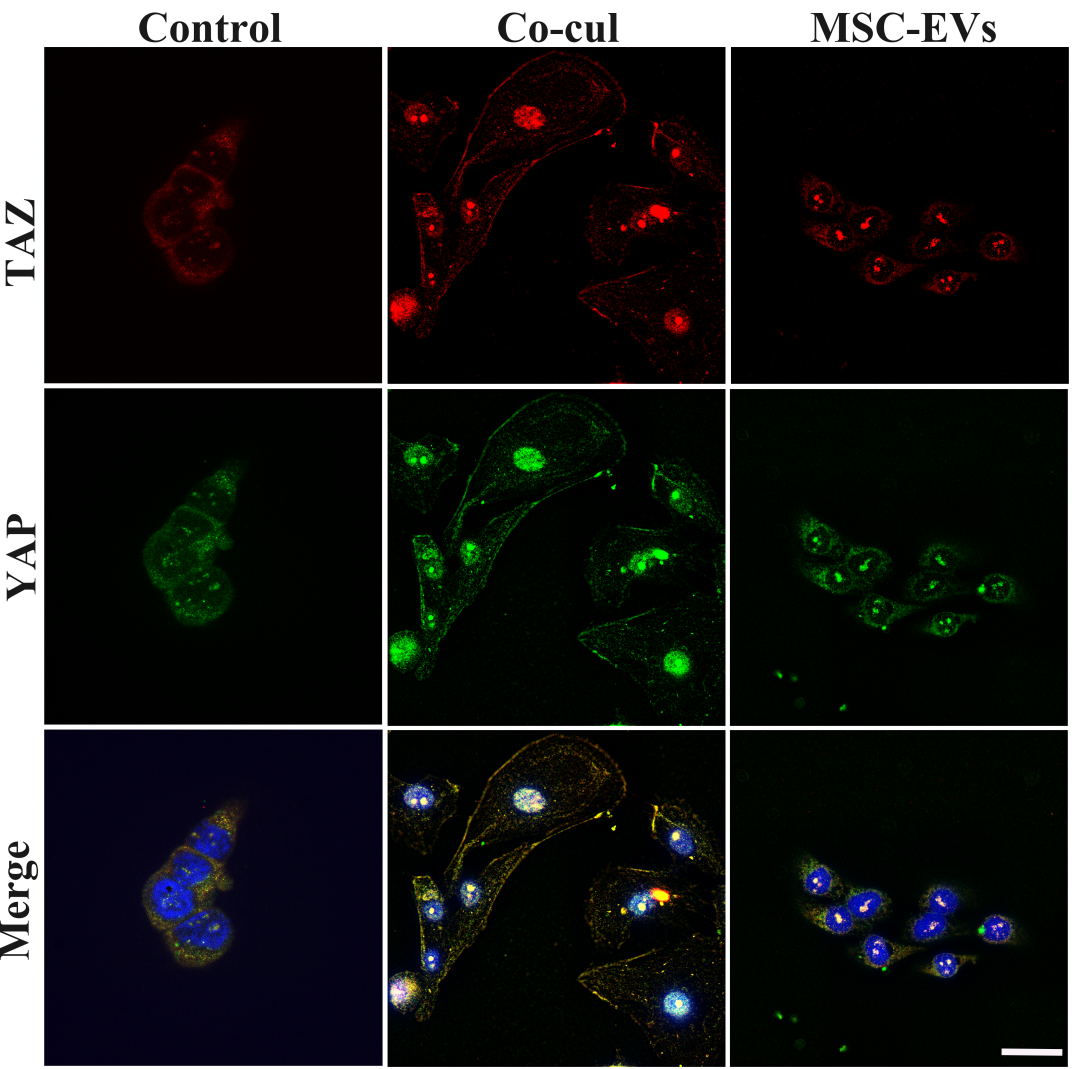


**Supplemental Figure 16. MSC-EVs inhibit the YAP/TAZ pathway.** Representative immunofluorescence staining images of TAZ and YAP in NRK-52E after MSC-EVs intervention (Scale bar: 100 μm);


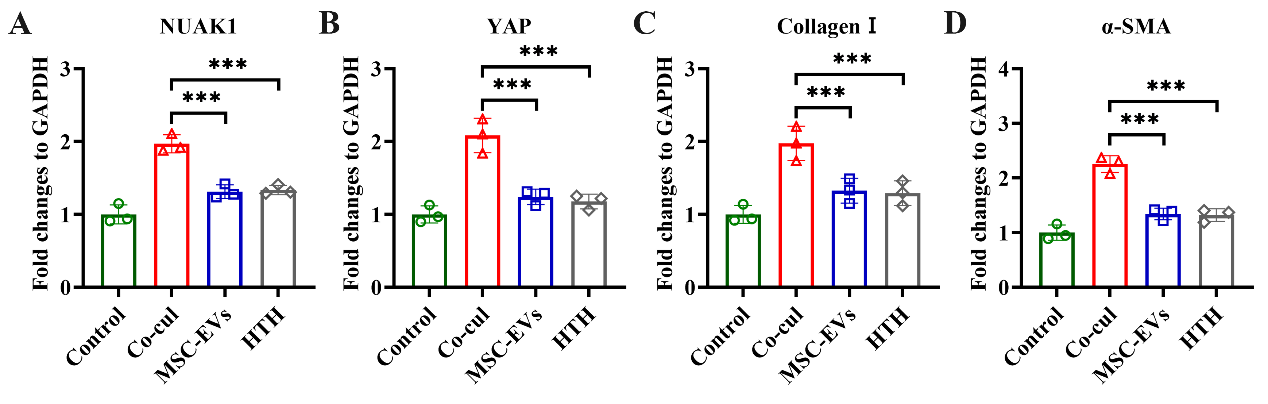


**Supplemental Figure 17.** Quantitative analysis of Western blot bands shown in Figure 3C (n=3). All values are presented as the mean ± SD. ****P* < 0.001.


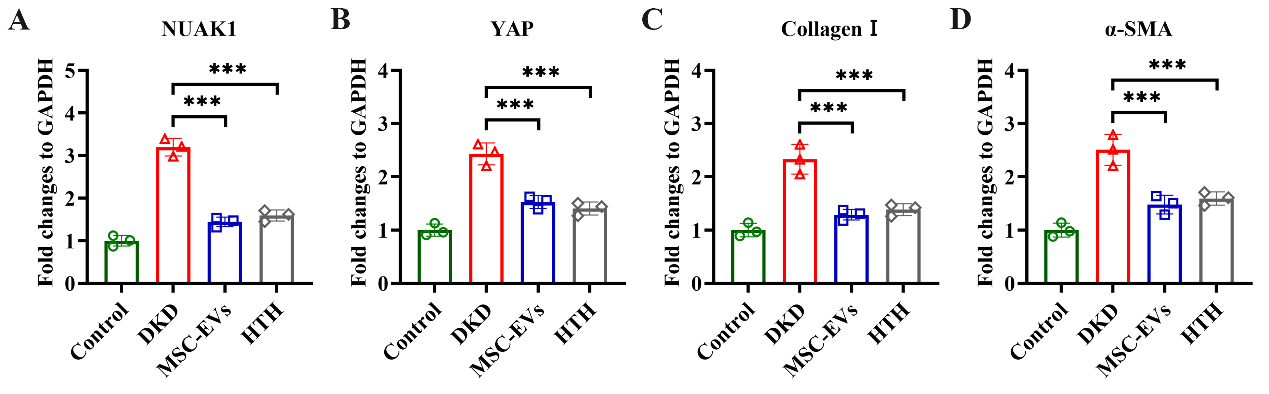


**Supplemental Figure 18.** Quantitative analysis of Western blot bands shown in Figure 3F (n=3). All values are presented as the mean ± SD. ****P* < 0.001.


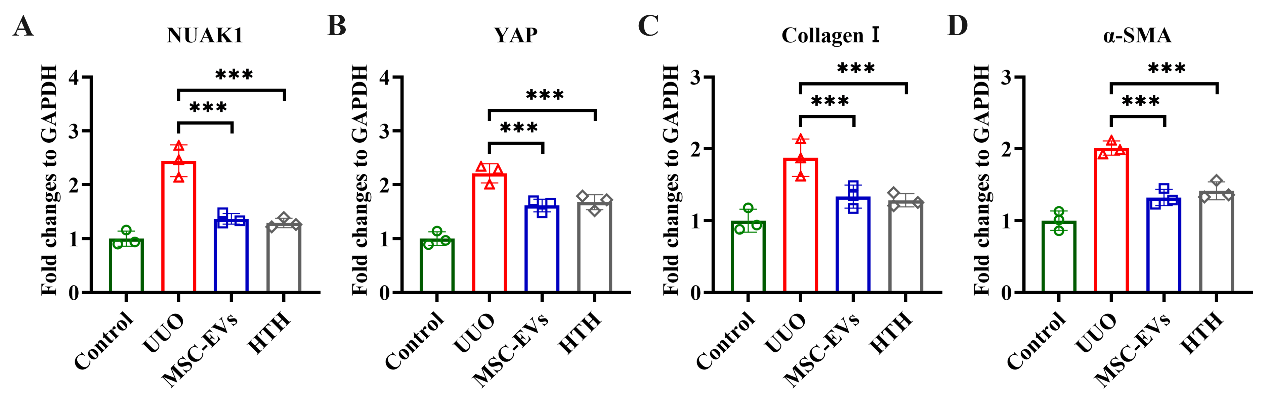


**Supplemental Figure 19.** Quantitative analysis of Western blot bands shown in Figure 3G (n=3). All values are presented as the mean ± SD. ****P* < 0.001.


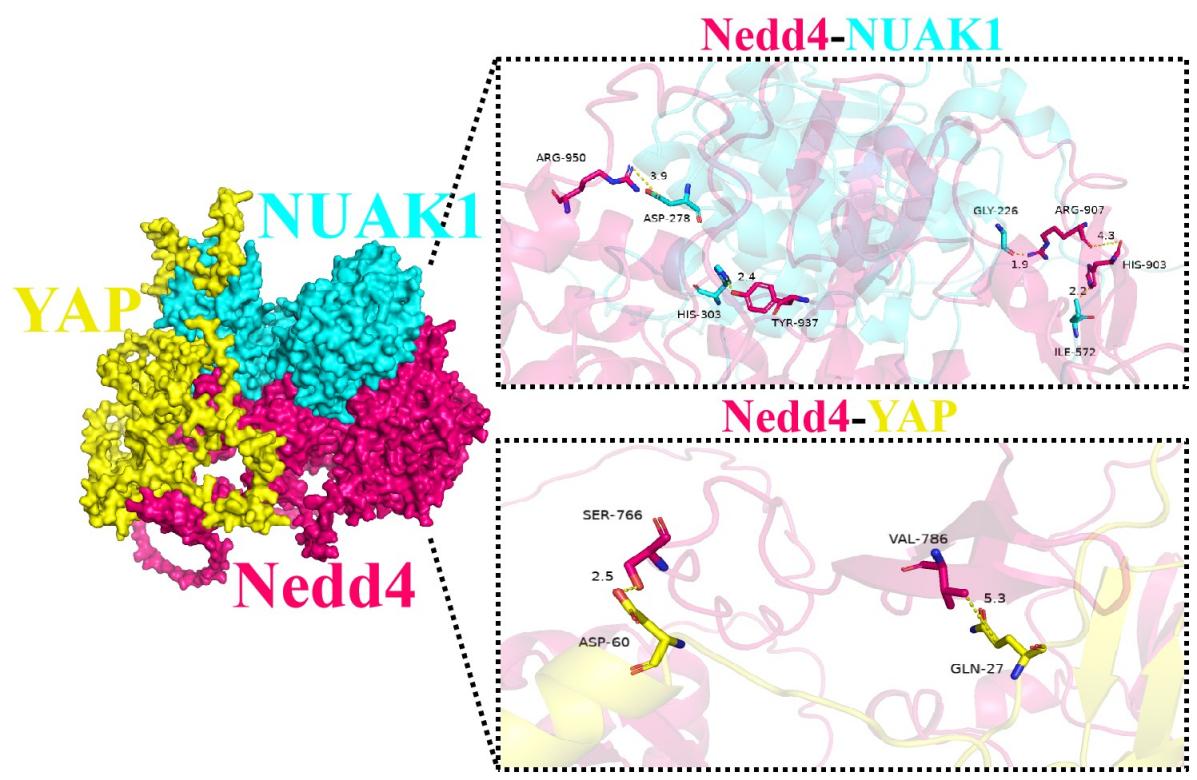


**Supplemental Figure 20. The docking model of Nedd4 with NUAK1 and YAP.**

Left panel: magnified view of NUAK1-YAP-Nedd4 binding. Right panel: Above, The pyridine ring of NUAK1 (HIS-303) forms a hydrogen bond force of 2.4 Å with the hydroxyl group of the side chain of Nedd4 (TYR-937). Both have a cyclic rigid planar structure and π-π stacking exists. The secondary amino group (ARG-950) of the Nedd4 side chain forms a hydrogen bond force of 3.9 Å with the carboxyl group (ASP-278) of NUAK1. Below, A 2.5 Å hydrogen bond is formed between the carboxyl groups of the side chains of Nedd4 (SER-766) and YAP (ASP-60). The alkyl main chain (GLN-27) of YAP forms a hydrophobic interaction of 5.3 Å with the hydrophobicity of Nedd4 (VAL-786).


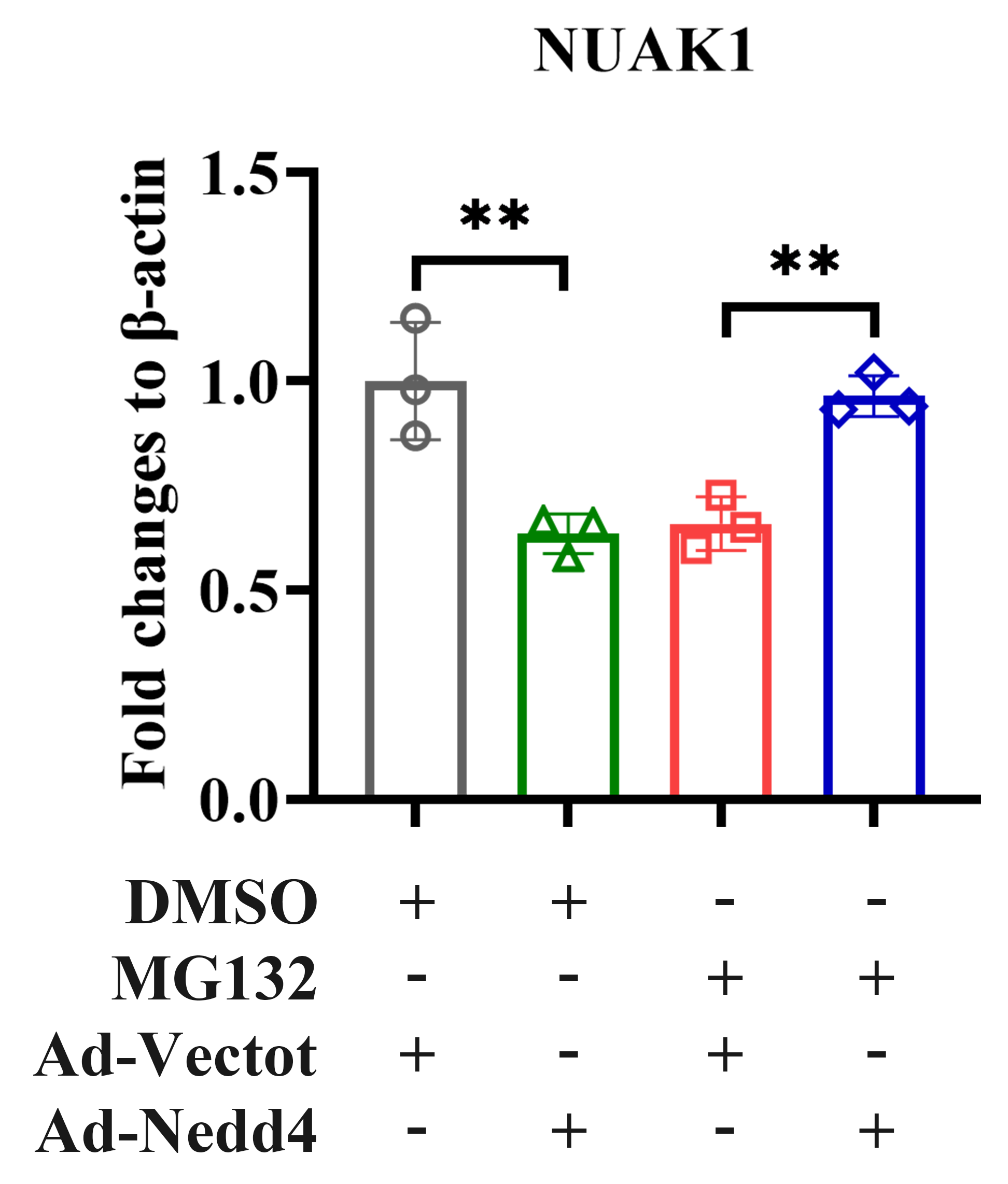


**Supplemental Figure 21.** Quantitative analysis of Western blot bands shown in Figure 4E (n=3). All values are presented as the mean ± SD. **P* < 0.05, ****P* < 0.001.


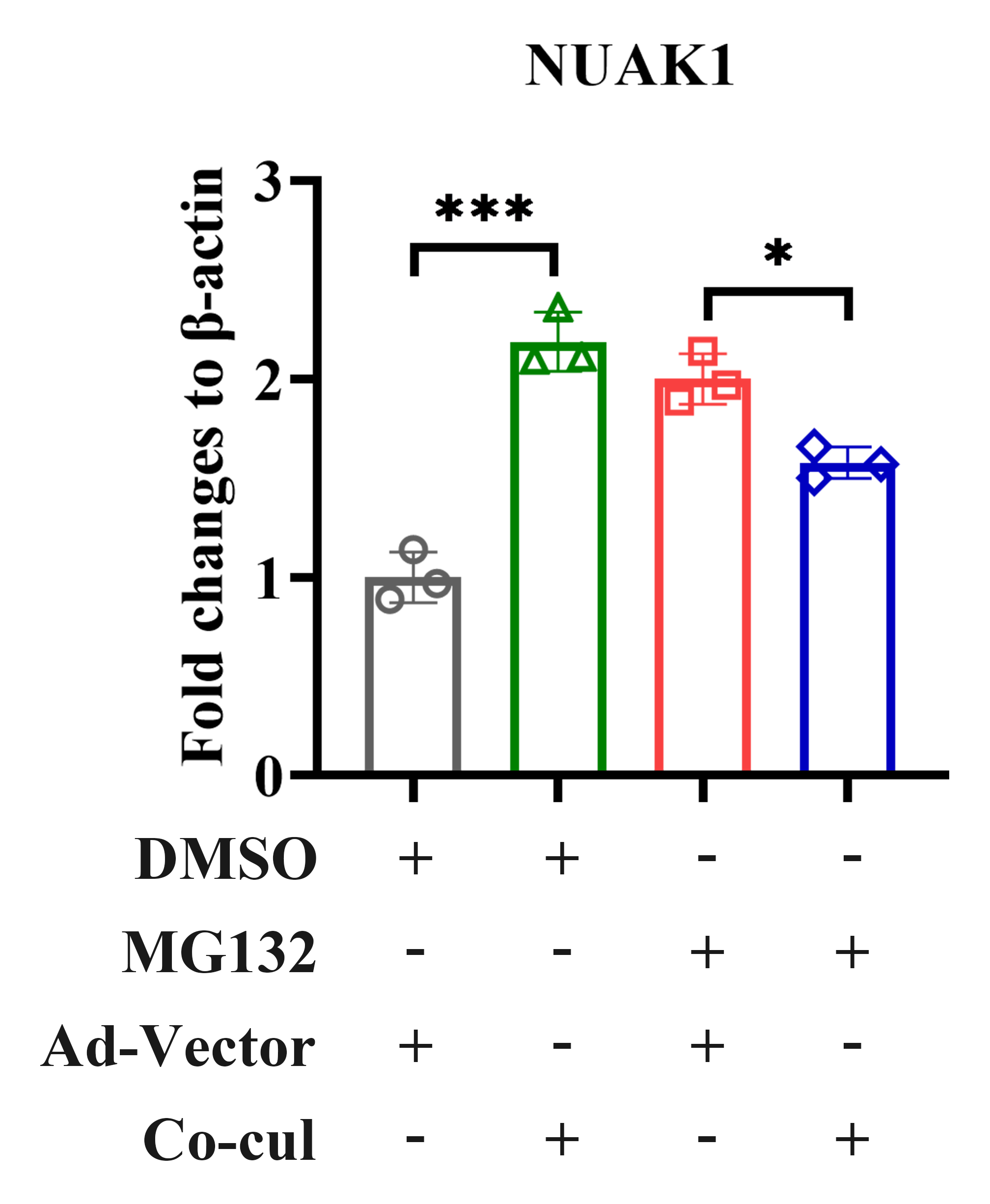


**Supplemental Figure 22.** Quantitative analysis of Western blot bands shown in Figure 4F (n=3). All values are presented as the mean ± SD. **P* < 0.05, ****P* < 0.001.


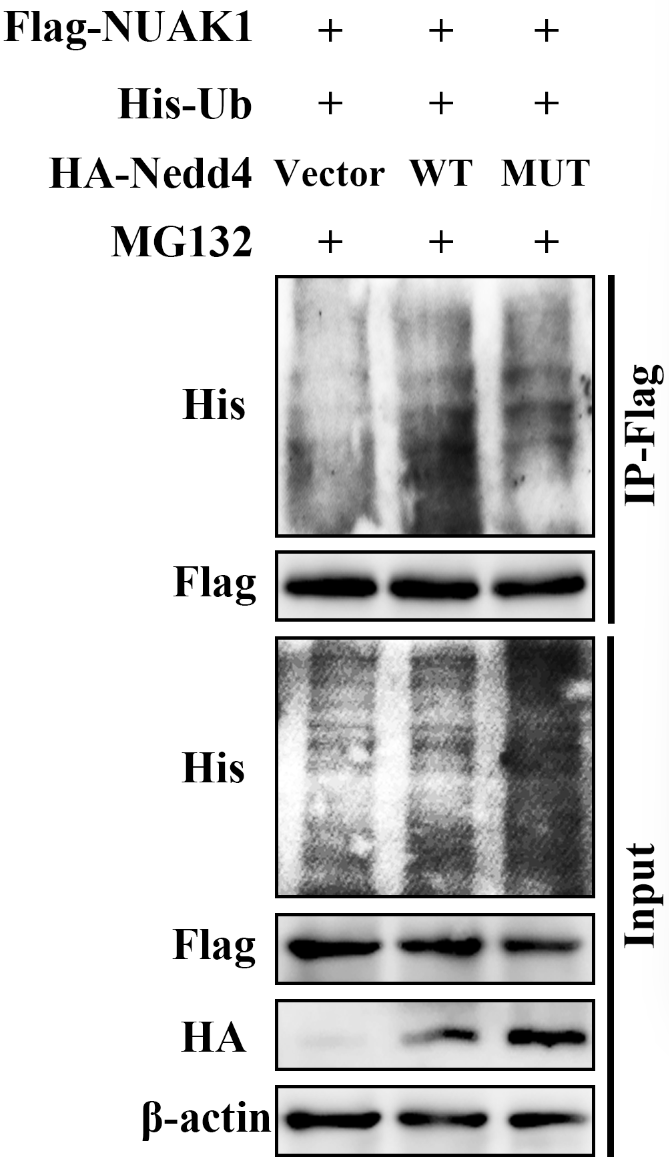


**Supplemental Figure 23.** Wild-type Nedd4 promotes NUAK1 ubiquitination in cells.

Cells were co-transfected with Flag-NUAK1, His-Ub, and either empty vector, HA-Nedd4-WT, or catalytically inactive HA -Nedd4 mutant, followed by MG132 treatment before harvest. Cell lysates were analyzed by immunoblotting (Input). Flag-NUAK1 was immunoprecipitated with anti-Flag, and ubiquitinated NUAK1 was detected by immunoblotting with anti- His. Wild-type Nedd4 markedly increased NUAK1 ubiquitination, whereas the catalytically inactive mutant showed a much weaker effect.


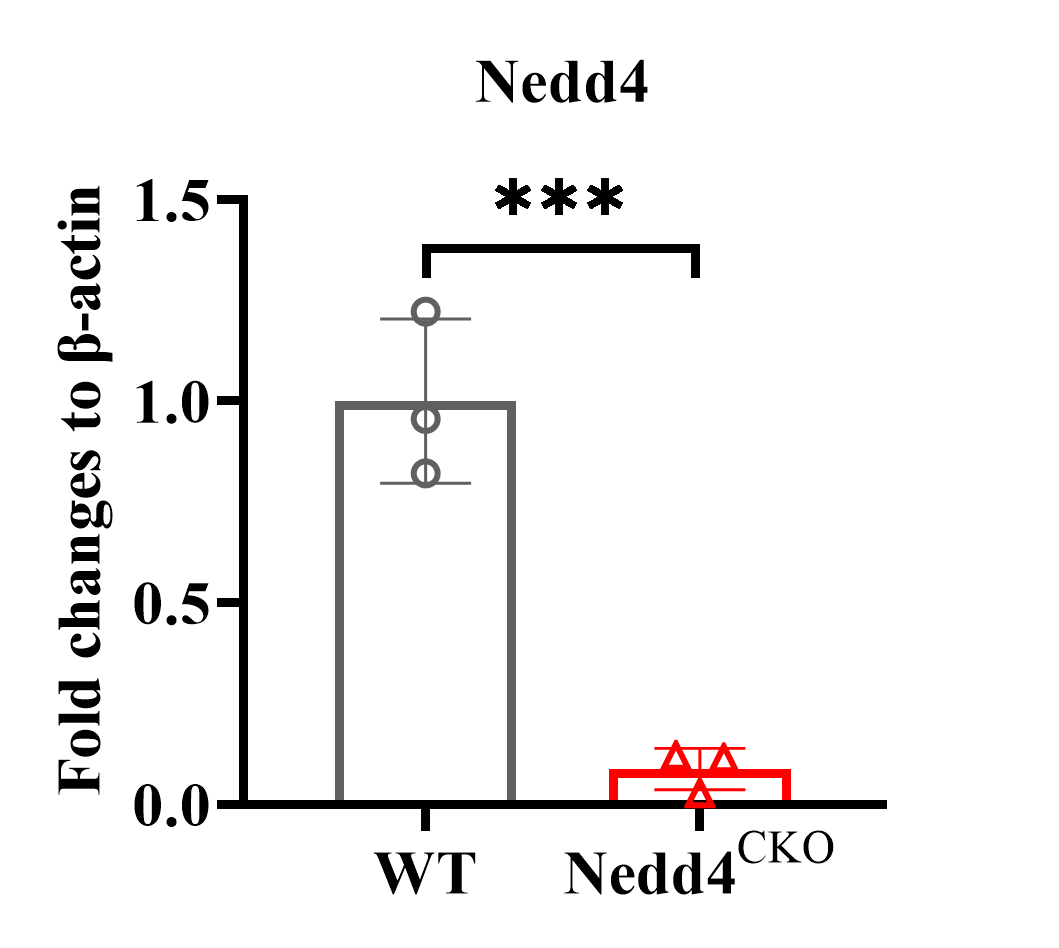


**Supplemental Figure 24.** Quantitative analysis of Western blot bands shown in Figure 5B (n=3). All values are presented as the mean ± SD. ****P* < 0.001.


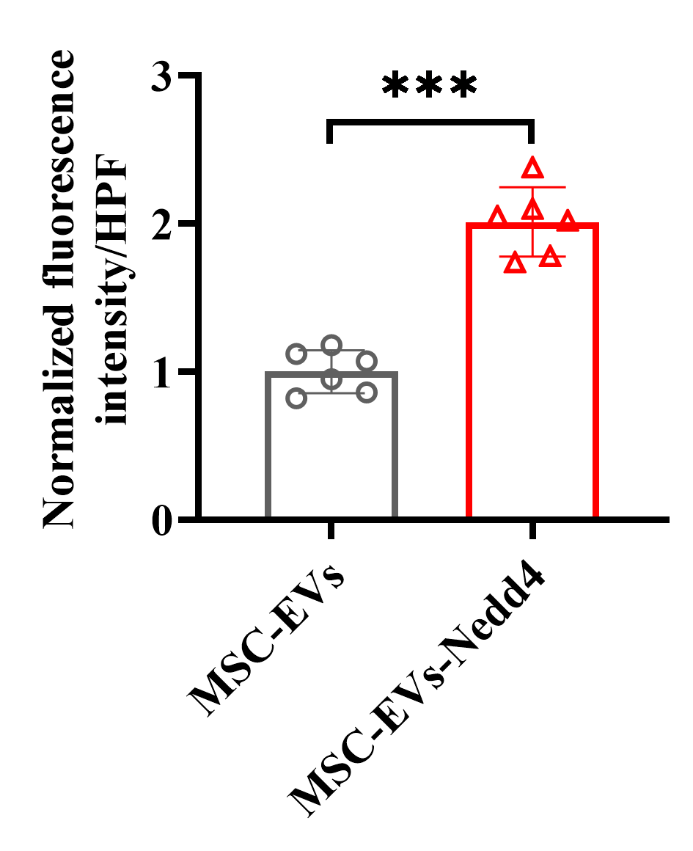


**Supplemental Figure 25.** Quantitative analysis of fluorescence intensity in Figure 6G (n = 6). All values are presented as the mean ± SD. ****P* < 0.001.


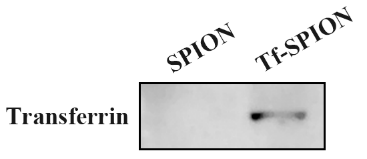


**Supplemental Figure 26.** Western blot showing Tf in Tf-SPIONs.


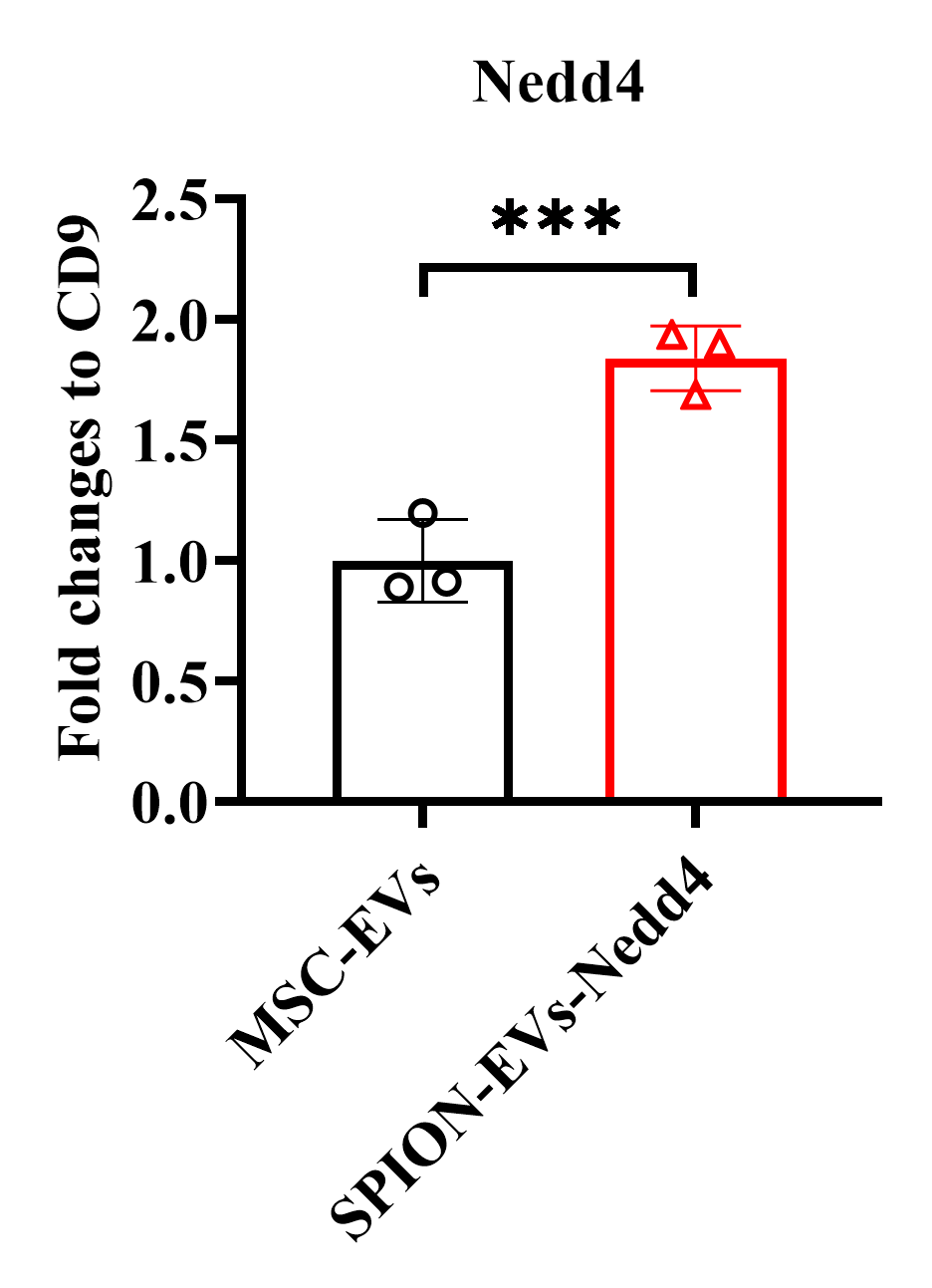


**Supplemental Figure 27.** Quantitative analysis of Western blot bands shown in Figure 6J (n=3). All values are presented as the mean ± SD. ****P* < 0.001


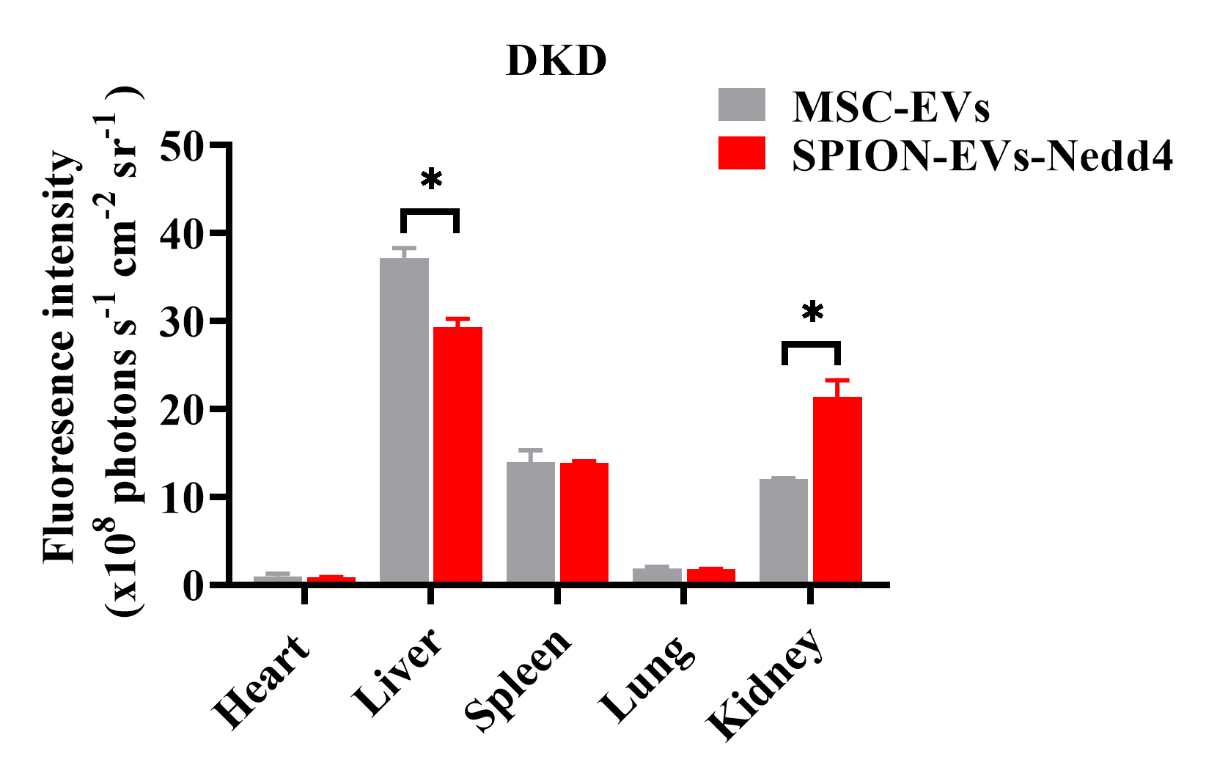


**Supplemental Figure 28.** Fluorescence intensity of heart, liver, spleen, lungs, and kidneys (n=3). All values are presented as the mean ± SD. **P* < 0.05.


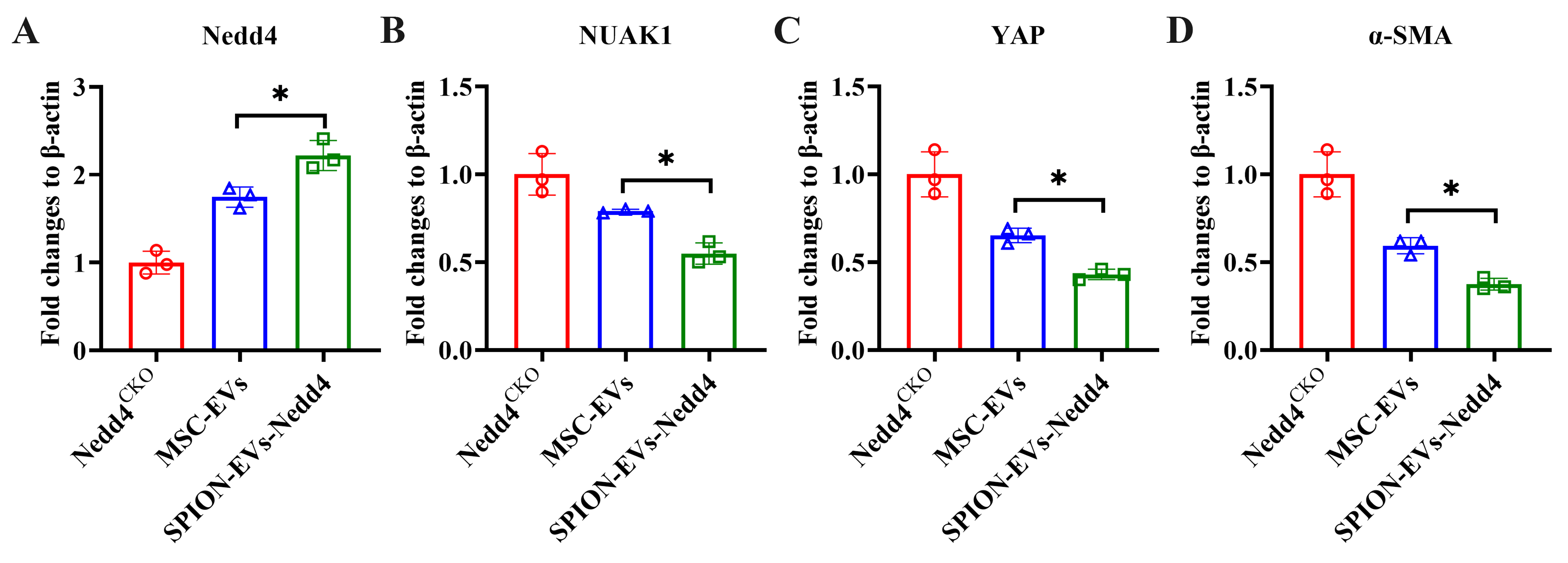


**Supplemental Figure 29.** Quantitative analysis of Western blot bands shown in Figure 7F (n=3). All values are presented as the mean ± SD. **P* < 0.05.

**
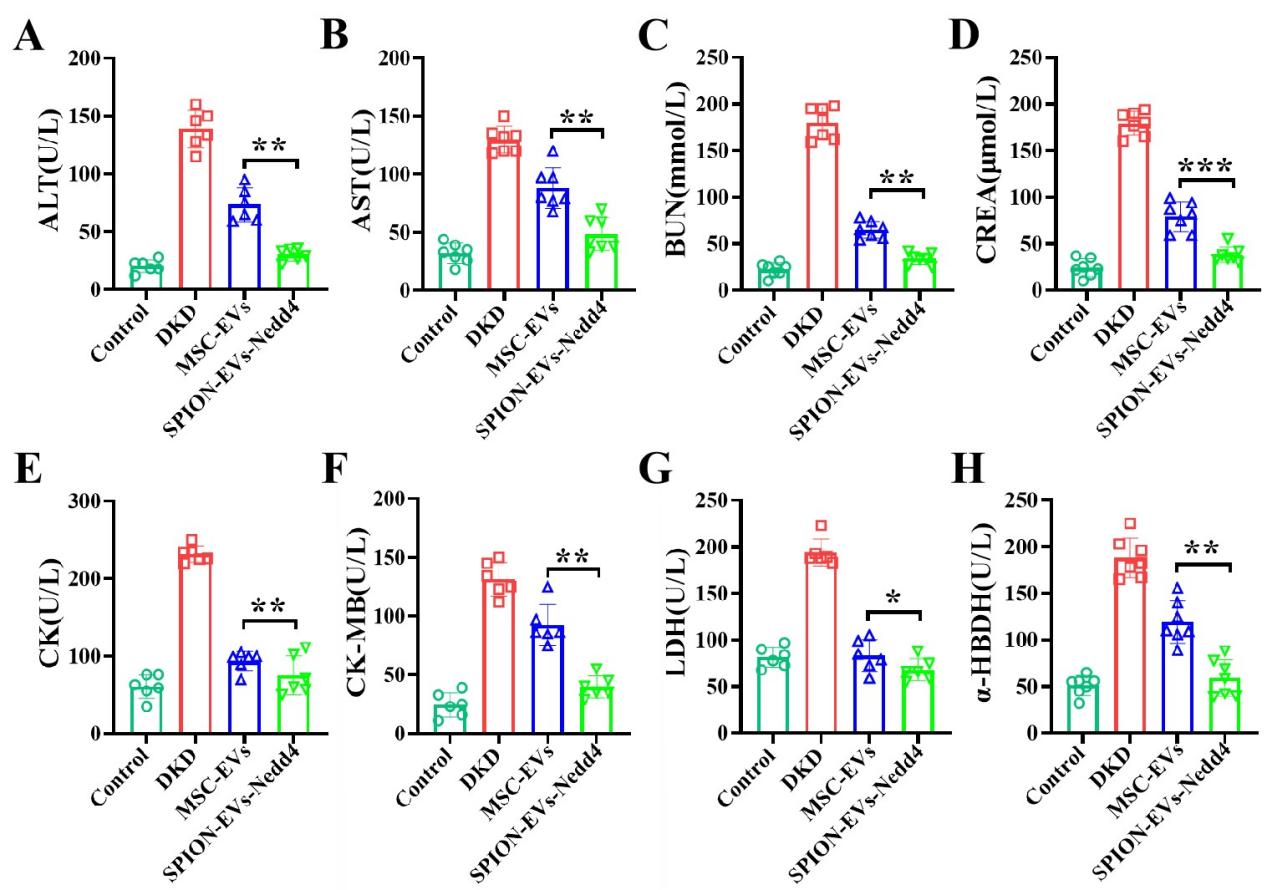
**

**Supplemental Figure 30. Safety of dual-targeted engineered SPION-EVs-Nedd4 in DKD model.** A-D. The expression levels of alanine aminotransferase (ALT), aspartate aminotransferase (AST), blood urea nitrogen (BUN) and creatinine (CREA) were determined by biochemical tests. E-H. The expression levels of creatine kinase (CK), creatine kinase isoenzyme (CK-MB), lactate dehydrogenase (LDH), and α-hydroxybutyrate dehydrogenase (α-HBDH) were examined by blood biochemical tests. All values are presented as the mean ± SD. **P* < 0.05, and ***P* < 0.01, ****P* < 0.001.


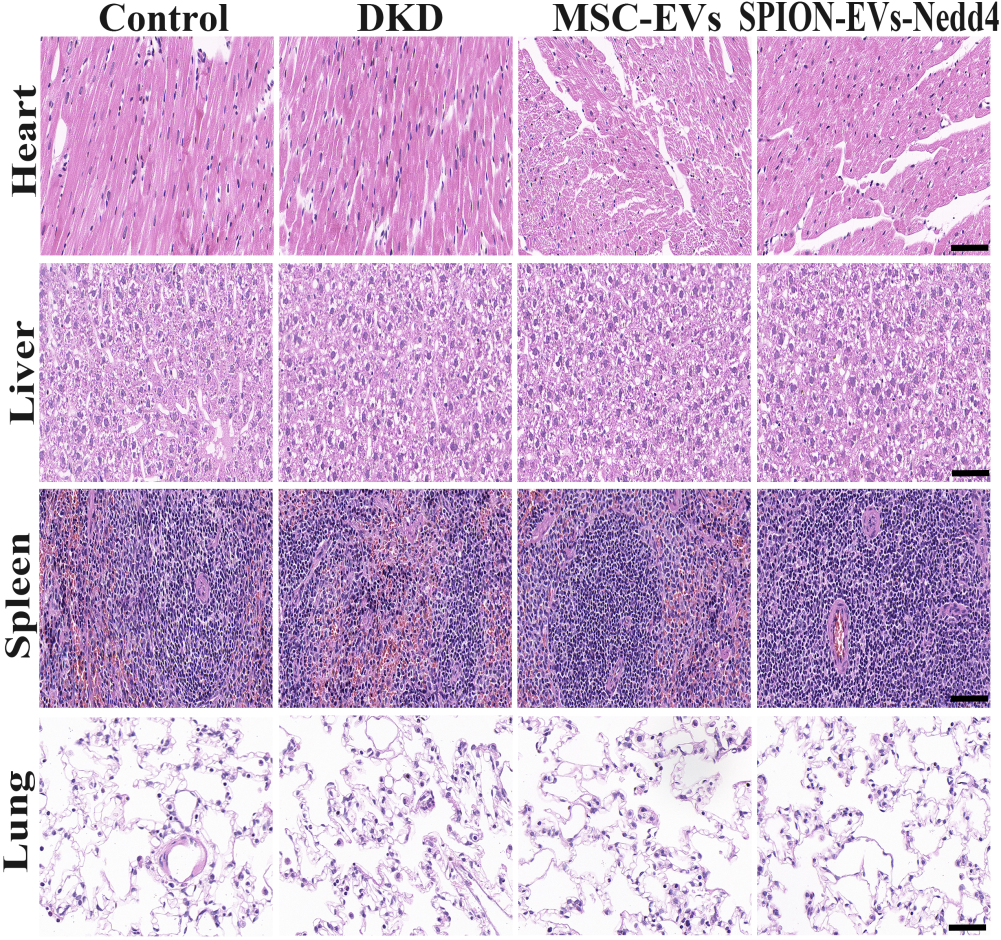


**Supplemental Figure 31.** Histopathological HE staining results of organs such as the heart, liver, spleen and lungs (Scale bar: 100 μm).


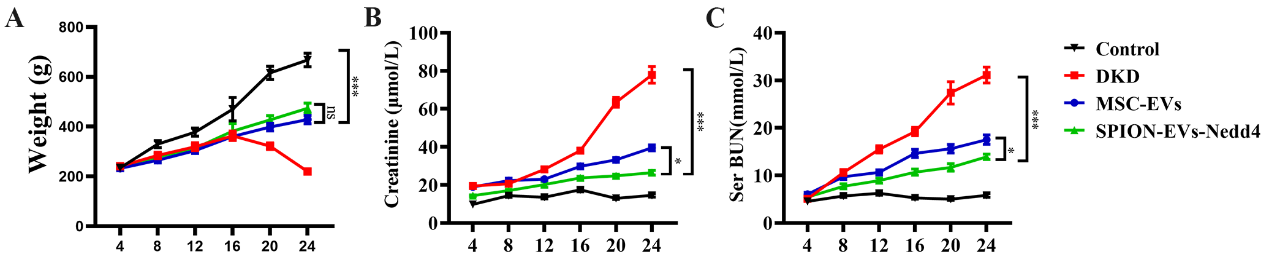


**Supplemental Figure 32.** Long-term biosafety assessment of engineered EVs. A. The impact of engineered EVs on weight; B. The impact of engineered EVs on creatinine levels; C. The impact of engineered EVs on BUN. All values are presented as the mean ± SD. **P* < 0.05, ****P* < 0.001.
